# Chicken telomerase reverse transcriptase promotes the tumorigenicity of avian leukosis virus subgroup J by regulating the Wnt/β-catenin signaling pathway

**DOI:** 10.1186/s13567-022-01120-2

**Published:** 2022-12-02

**Authors:** Yong Xiang, Canxin Liang, Qingbo Li, Qinxi Chen, Yang Zhou, Xiaoxue Zheng, Di Zhou, Zepeng Wang, Guyao Wang, Weisheng Cao

**Affiliations:** 1 College of Veterinary Medicine, South China Agricultural University, Guangzhou, 510642 China; 2 Key Laboratory of Zoonosis Prevention and Control of Guangdong Province, South China Agricultural University, Guangzhou, 510642 China; 3 National and Regional Joint Engineering Laboratory for Medicament of Zoonosis Prevention and Control, South China Agricultural University, Guangzhou, 510642 China; 4 Key Laboratory of Zoonosis of the Ministry of Agriculture and Rural Affairs, South China Agricultural University, Guangzhou, 510642 China; 5 Key Laboratory of Veterinary Vaccine Innovation of the Ministry of Agriculture and Rural Affairs, South China Agricultural University, Guangzhou, 510642 China

**Keywords:** Avian leukosis virus subgroup J, chicken telomerase reverse transcriptase, Wnt/β-catenin signaling pathway, tumorigenicity, in vivo

## Abstract

This research aimed to analyze the regulatory effect of chicken telomerase reverse transcriptase (chTERT) on the Wnt/β-catenin signaling pathway and its effect on the tumorigenicity of avian leukosis virus subgroup J (ALV-J) through in vivo experiments. The chTERT eukaryotic expression plasmid and its recombinant lentivirus particles were constructed for in vivo transfection of chTERT to analyze the effect of chTERT continuously overexpressed in chickens on the tumorigenicity of ALV-J. During 156 days of the artificial ALV-J tumor-inducing process, 7 solid tumors developed in 3 chickens in the chTERT-overexpression group (*n* = 26*2) and no tumors developed in the control group (*n* = 26*2). Another 18 tumors induced by ALV-J were confirmed and collected from breeding poultry farms. And we confirmed that chTERT was significantly highly expressed in ALV-J tumors. The ELISA data suggested that the protein levels of β-catenin and c-Myc in the chicken plasma of the chTERT-overexpressing group with ALV-J infected were consistently and significantly higher than those of the control group. Compared with that of the tumor-adjacent tissues, the activity of the Wnt/β-catenin signaling pathway and expression of the c-Myc was significantly increased in ALV-J tumors. And the percentage of apoptosis in ALV-J tumors significantly lower than that in tumor-adjacent tissues. Immunohistochemistry, Western blot and RT-qPCR suggested that the replication level of ALV-J in tumors was significantly higher than that in tumor-adjacent tissues. This study suggests that chTERT plays a critical role in the tumorigenicity of ALV-J by enhancing the Wnt/β-catenin signaling pathway, which will contribute to further elucidating the tumor-inducing mechanism of ALV-J.

**Supplementary Information:**

The online version contains supplementary material available at 10.1186/s13567-022-01120-2.

## Introduction

Avian leukosis (AL) is a general term for a variety of tumor diseases in poultry caused by avian leukosis virus (ALV) [1, 2]. This avian disease causes a decrease in the egg production rate, a decrease in egg quality and carcass quality, and an increase in the mortality rate [3]. This disease is found worldwide, affecting almost all breeding and commercial flocks, mainly causing tumors and immunosuppression in infected chickens [4, 5]. Since the 1990 s, AL, especially avian leukosis virus subgroup J (ALV-J), has caused major economic losses to China’s chicken industry [6], which has seriously harmed the healthy development of white-feathered broilers, egg-type chickens, yellow-feathered broilers and local chickens [7–9]. AL is a class II animal disease that the Ministry of Agriculture and Rural Affairs of China prioritized for prevention and control and a germline disease that should be eliminated in the National Plan for Genetic Improvement of Broilers (2014–2025). Prevention and control of ALV should be strengthened, and its pathogenic mechanism should also be actively explored to provide a theoretical basis for scientific prevention and control measures of ALV.

Telomerase reverse transcriptase (TERT) is the core component that ensures the normal functioning of telomerase [10–12], and its expression has a positive effect on telomerase activity [13, 14]. Human TERT (hTERT) activation plays a key role in the process of telomere extension and participates in the growth and development of cells and the development of tumors [13, 15, 16]. Transcriptional reactivation of hTERT in various human malignant tumors is closely regulated by multiple signaling pathways, among which the Wnt/β-catenin signaling pathway is one of the most relevant [17–20]. For example, in nasopharyngeal carcinoma, a bidirectional positive feedback loop between hTERT and the Wnt/β-catenin signaling pathway, through which the characteristics of cancer stem cells in radiation-resistant nasopharyngeal carcinoma cells are regulated, was identified [21]. In keloids, the Wnt/β-catenin signaling pathway was shown to enhance cell proliferation and migration by regulating hTERT [18].

A previous study by our team showed that the insertion and integration of the proviral cDNA of chronic transformed ALV-J near the host chicken telomerase reverse transcriptase (chTERT) gene was weakly selective during the long tumor-inducing process [22]. In addition, another previous study by our team indirectly revealed the potential role and mechanism of chTERT in ALV-J tumor induction at the cellular level, namely, that chTERT protein and the Wnt/β-catenin signaling pathway can regulate each other to inhibit apoptosis, promote cell proliferation and migration in LMH cells. The chTERT can also upregulate the replication level of chronic transformation ALV-J and the expression level of the proto-oncogene c-Myc [14]. To obtain more powerful evidence to support the above conclusions, we aimed to perform animal experiments. Therefore, the primary purpose of this study was to intuitively analyze the effect of chTERT on ALV-J tumorigenicity after overexpression of chTERT in chickens. Second, the tumor tissues, tumor-adjacent tissues of ALV-J and corresponding normal tissue were used as the main samples to analyze the regulatory effect of the chTERT mediated Wnt/β-catenin signaling pathway and its impact on host cell growth and virus replication. Thus, our results will help elucidate the role and molecular mechanism of chTERT in chronic transformed ALV-J tumor induction.

## Materials and methods

### Cells, viruses and antibodies

The 293 T, LMH and DF-1 cell lines were kept in our laboratory. The 293 T and DF-1 cell lines were cultured in dulbecco’s modified eagle medium (DMEM), and LMH cells were cultured in DMEM/F12 (Gibco, Thermo Fisher Scientific, Inc.) with 10% fetal bovine serum (FBS) and maintained at 37 °C with 5% CO_2_. The chronic transformed ALV-J Hc1 strain was also kept in our laboratory.

The primary antibodies of β-catenin and c-Myc were the products of Santa Cruz Biotechnology (Santa Cruz, CA, USA). The primary antibodies of TCF4 and Cyclin D1 were the products of Affinity Biosciences, Ltd. (USA). An GAPDH primary antibody was the product of Abcam, Inc. (UK). An eGFP-Tag primary antibody was the product of Invitrogen, Thermo Fisher Scientific, Inc. (California, USA). A FLAG-Tag primary antibody was the product of Sigma-Aldrich, St. Louis, Missouri (USA). The mouse monoclonal antibodies G2.3 and JE9 against the gp85 protein of ALV-J was kindly provided by the Avian Disease and Oncology Laboratory (USA) and Professor Aijian Qin of Yangzhou University, respectively. The single factor serum of chTERT protein was prepared and preserved by our laboratory in the early stage.

### Plasmids

The coding region sequence of the chTERT gene and protein (GenBank: NM_001031007.1, UniProt: Q6RD80_CHICK) was optimized for chicken preference and synthesized by Sangon Biotech (Shanghai, China). We used the pLV-sfGFP(2 A) (eGFP green fluorescence) lentiviral expression vector and pCAGGS eukaryotic expression vector, combined with homologous recombination, to link the codon-optimized chTERT gene sequence to the above vector [14]. The chTERT gene was labeled with a FLAG and eGFP tag in the pLV-sfGFP(2 A) vector and pCAGGS vector, respectively. Finally, the pLV-chTERT-FLAG and pCAGGS-chTERT-eGFP plasmids, as well as their negative control plasmids (pLV-NC and pCAGGS-eGFP) were obtained.

### Artificial tumorigenic experiment of ALV-J

**Figure 1:**
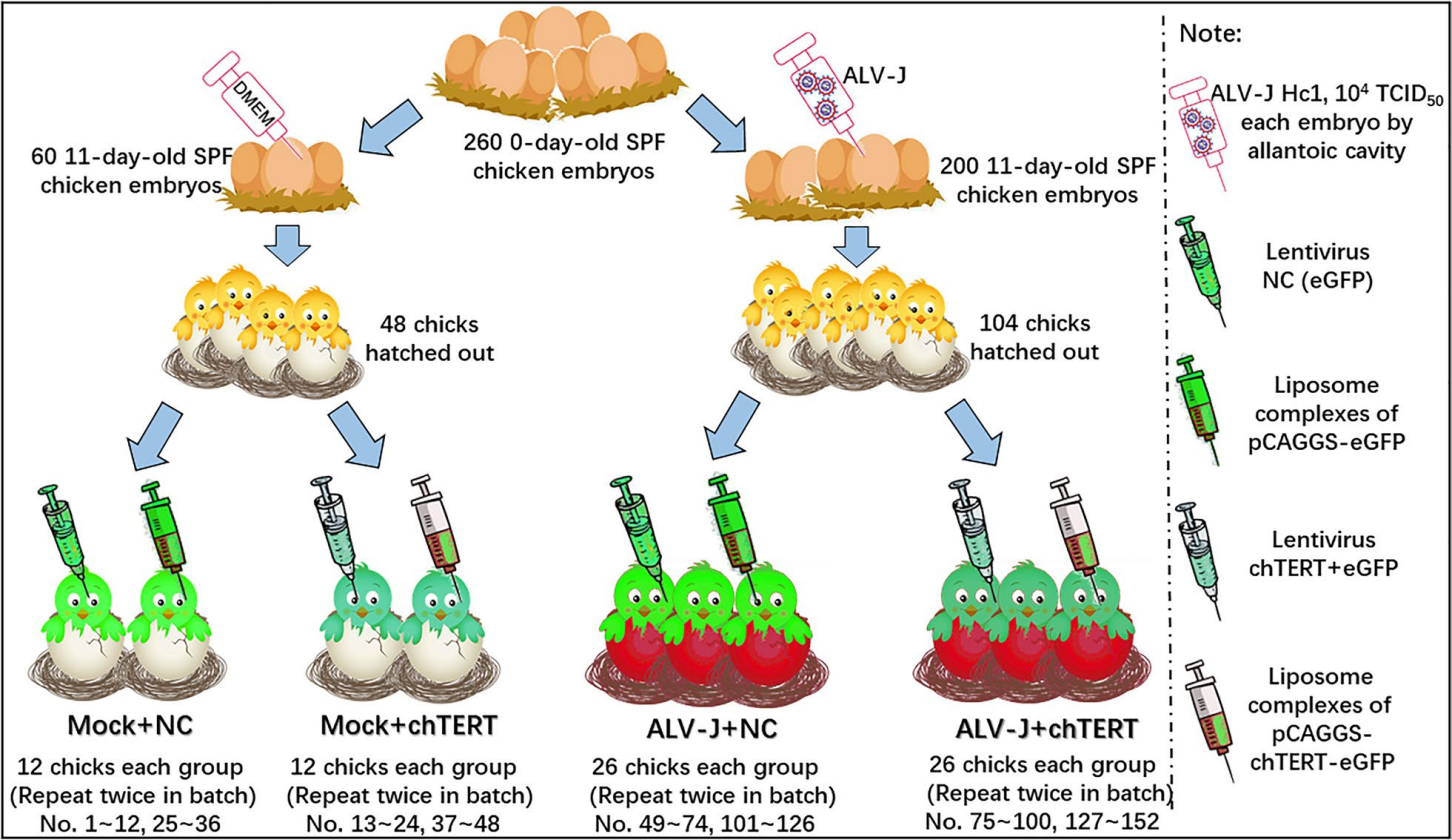
**
Design of ALV-J artificial tumor animal experiment.** In vivo transfection was performed after chicks hatched, in which the liposome complexes of eukaryotic expression plasmids (pCAGGS-chTERT-eGFP, pCAGGS-eGFP) were injected intramuscularly and intraperitoneally, 200 µg per chicken, with supplementary injection every 2 weeks. The recombinant lentivirus (lentivirus chTERT + eGFP, lentivirus NC) was injected intraperitoneally, 200 µL (10^5^ TU·mL^-1^) per chicken, with supplementary injection every 1 month. Two intra-batch repeats were performed for each group.

A total of 260 0-day-old specific pathogen-free (SPF) chicken embryos were obtained from Jinnan Sais Poultry Co., Ltd. (Jinan, China). The animal experimental design is shown in Figure 1. This study combined lentiviral infection and eukaryotic expression plasmid injection for in vivo transfection of chTERT gene in chickens after hatch out. The packaging of the lentivirus was carried out following the user manual for the lentiviral gene overexpression system (Invivogen, Wuhan, China) [14], and the lentiviruses (chTERT and NC) were then used to infect chickens. Polyethylenimine (PEI) (Sigma-Aldrich, St. Louis, Missouri, USA) was used as the in vivo transfection reagent for eukaryotic expression plasmids (pCAGGS-chTERT-eGFP and pCAGGS-eGFP), which can form liposome complexes with PEI to achieve transfection. Notably, the chTERT expression validation in vitro was performed before transfection in vivo.

### Collection of tissue samples

In necropsy examination of chickens with suspected tumor incidence, the central site of solid tumor occurrence was collected as tumor tissue in aseptic operation. Meanwhile, the site about 1–2 cm away from the tumor center and without solid tumor was taken as the corresponding tumor-adjacent tissue, and the site of healthy chickens at the same age was taken as its normal tissue. The size of the tissue blocks was less than 0.4 × 0.4 × 0.4 cm, and the tissue blocks were put into a 1.5 mL centrifuge tube. The tissue blocks were frozen in liquid nitrogen for 3–4 h, then moved to a −80 ℃ refrigerator for long-term storage. Samples were used for nucleic acid and protein extraction, etc. In addition, another sample of tumor tissue, tumor-adjacent tissue and normal tissue were taken. The blood on the tissue was cleaned with phosphate buffered saline (PBS), and placed in a 5 mL centrifuge tube filled with 4% paraformaldehyde. Samples were stored at room temperature for HE staining, frozen section, immunohistochemistry, TUNEL assay, etc.

### Real-time fluorescence quantitative PCR (RT-qPCR)

The tissue samples were homogenized by a cryogenic grinder, and then, the total RNA was extracted from tissues according to the manufacturer’s recommendations for TRIzol reagent (Fastagen Biotech, Shanghai, China). The total RNA was reverse transcribed to cDNA using a PrimeScript RT Reagent kit (TaKaRa, Japan), followed by RT-qPCR experiments, which were performed with Hieff® qPCR SYBR Green Master Mix (Yeasen, Shanghai, China) on a CFX96TM Real-time fluorescence quantitative PCR System (Bio-Rad, California, USA). The mRNA levels of target genes were quantified following the 2^−ΔΔCt^ method, and normalized to the GAPDH gene expression level [23]. The primers used were the same as those used in our previous study [14].

### Western blotting

The tissue samples were homogenized at low temperature, after which NP40 lysis buffer (Beyotime, Shanghai, China) was used to extract protein. The concentration of extracted protein was determined by the BCA Protein Assay Kit (Beyotime, Shanghai, China). Subsequently, the same amounts of protein in cell lysate were divided by SDS-PAGE buffer (Beyotime, Shanghai, China), and then transferred onto a nitrocellulose membrane. The different membranes were immunoblotted with different primary antibodies at 4 °C for 12–18 h. After that, the membranes were immunoblotted with the corresponding secondary antibody at 37 °C for 1 h. The blots were scanned through the Odyssey Infrared Imaging System (LI-COR, Nebraska, USA). When analyzing the results, Image J software was used to analyze the gray value of the protein band, calculate the ratio of the gray value of the target protein to the GAPDH protein, and then normalize the ratio of the different experimental groups.

### Enzyme-linked immunosorbent assay (ELISA)

The level of the ALV p27 protein in the supernatant of cell culture or plasma was measured with an Avian Leukosis Virus Antigen Test Kit (IDEXX Laboratories Pty., Ltd., Westbrook, USA) [24]. The levels of the β-catenin and c-Myc antigens in plasma were measured with a chicken-specific ELISA kit for β-catenin or c-Myc (Mlbio, Shanghai, China) following the manufacturer’s protocol.

### Frozen sections

During in vivo transfection, both the recombinant lentivirus of chTERT gene and its eukaryotic expression plasmid were labeled with green fluorescence (eGFP). Therefore, the fluorescence of the tissue samples was directly observed through frozen sections to confirm the successful overexpression of chTERT in chickens. Briefly, the specimen was immersed in Tissue-Tek medium in a freezing microtome at −28 °C to obtain histological sections of 4–5 μm. Then, the sample was observed with a fluorescence microscope. Images of different frozen sections were captured by a Nikon Ti2 microscope system.

### Hematoxylin and eosin (HE) staining

The diseased chickens suspected of having tumors were dissected, and the tumor tissues were taken for HE staining to analyze the histopathological characteristics. The tissue samples were soaked in 4% paraformaldehyde for at least 4 h and then transferred to 70% ethanol. Individual lobes of the sample biopsy material were placed in processing cassettes, dehydrated through a serial alcohol gradient, and embedded in paraffin wax blocks. Before immunostaining, 5-µm-thick tissue sections were dewaxed in xylene, rehydrated through decreasing concentrations of ethanol, washed in PBS, and then stained with hematoxylin and eosin. After staining, sections were dehydrated through increasing concentrations of ethanol and xylene.

### Isolation and identification of ALV-J

Eighty microliters of plasma or grinding fluid of tumor tissue was inoculated into DF-1 cells (24-well plate), and negative and positive controls were established. After culture for 24 h in DMEM containing 10% FBS at 37 °C with 5% CO_2_, the cell culture medium was replaced with DMEM containing 1% FBS and maintained for 7–9 days. The supernatant was taken for ALV p27 detection by ELISAs. Moreover, the DNA of the cells or tumor tissues was extracted according to the instructions of the DNA extraction kit (OMEGA, USA) using previously reported primers to distinguish viral subgroups by RT-qPCR [25]. In addition, immunofluorescence and immunohistochemistry analysis was performed with an ALV-J monoclonal antibody (JE9) used for final confirmation.

### Immunohistochemical analysis

Tissue samples embedded in paraffin with HE staining were directly used for immunohistochemical analysis. Antigens were unmasked by microwaving sections in 10 mmol·L^− 1^ citrate buffer, pH 6.0 (15 min), and immunostaining was performed using the avidin biotinylated enzyme complex method with antibodies against β-catenin, c-Myc or ALV-J and equivalent concentrations of polyclonal nonimmune IgG controls. After incubation with the appropriate biotin-conjugated secondary antibody and then with streptavidin solution, color development was performed using 3,3-diaminobenzidine (DAB), and counterstaining was performed using hematoxylin. The images of sections were captured under a microscope, and the cells marked in brown were positive cells. Image J software can be used for relative quantification of the target protein; that is, at least 3 fields of view were randomly selected under high magnification, and with the assistance of Image J software, the pixels of the brown area in each field of view were quantified, and then, the ratio of the pixel area of the brown area to the total pixel area under the field was calculated, which was the positive ratio of the target protein.

### Terminal-deoxynucleotidyl transferase-mediated nick end labeling (TUNEL) assay

Paraffin-embedded sections of tissue samples were fixed in a freshly prepared paraformaldehyde solution for 5 min at room temperature. The fixed sections were washed with PBS for 5 min at room temperature and then treated with proteinase K in HCl (0.01 N, pH 2.0) for 15 min at room temperature. Then, the TUNEL reaction mixture of the One Step TUNEL Apoptosis Assay Kit (Beyotime, Shanghai, China) was added to each section, which was then coverslipped and incubated at 37 °C for 60 min. After the sections were washed with 0.2% Triton X-100 in PBS, they were incubated with Cy3-labeled secondary antibody, and DAPI was used for nuclear visualization. Images were captured using a Nikon Ti2 microscope and Nikon camera. Apoptotic cells are marked in red, and nuclei are marked in blue. Image J software can be used for relative quantification of positive cells in each experimental group; that is, at least 3 fields of view are randomly selected under a high magnification microscope. With the assistance of Image J software, the red cells and all blue nuclei in each field were counted and their ratio values were calculated, namely, the relative ratio of cell apoptosis in each experimental group.

### Telomerase activity assay

Telomerase activity was measured by the telomeric repeat amplification protocol (TRAP), which was carried out with the TeloTAGGG Telomerase PCR ELISA Kit (Sigma-Aldrich, St. Louis, Missouri, USA) following the manufacturer’s protocol [14].

### Statistical analysis

All the experiment data are presented as the mean ± standard deviation. The GraphPad Prism software was used to perform statistical analysis by Student’s *t* test, and it was considered significant difference when the *P* < 0.05. In this study, when the letters (a, b, c, d) corresponding to the error bars of each experimental group are different, it means that there is a significant statistical difference between them, that is, *P* < 0.05.

## Results

### chTERT is continuously overexpressed in chickens

**Figure 2:**
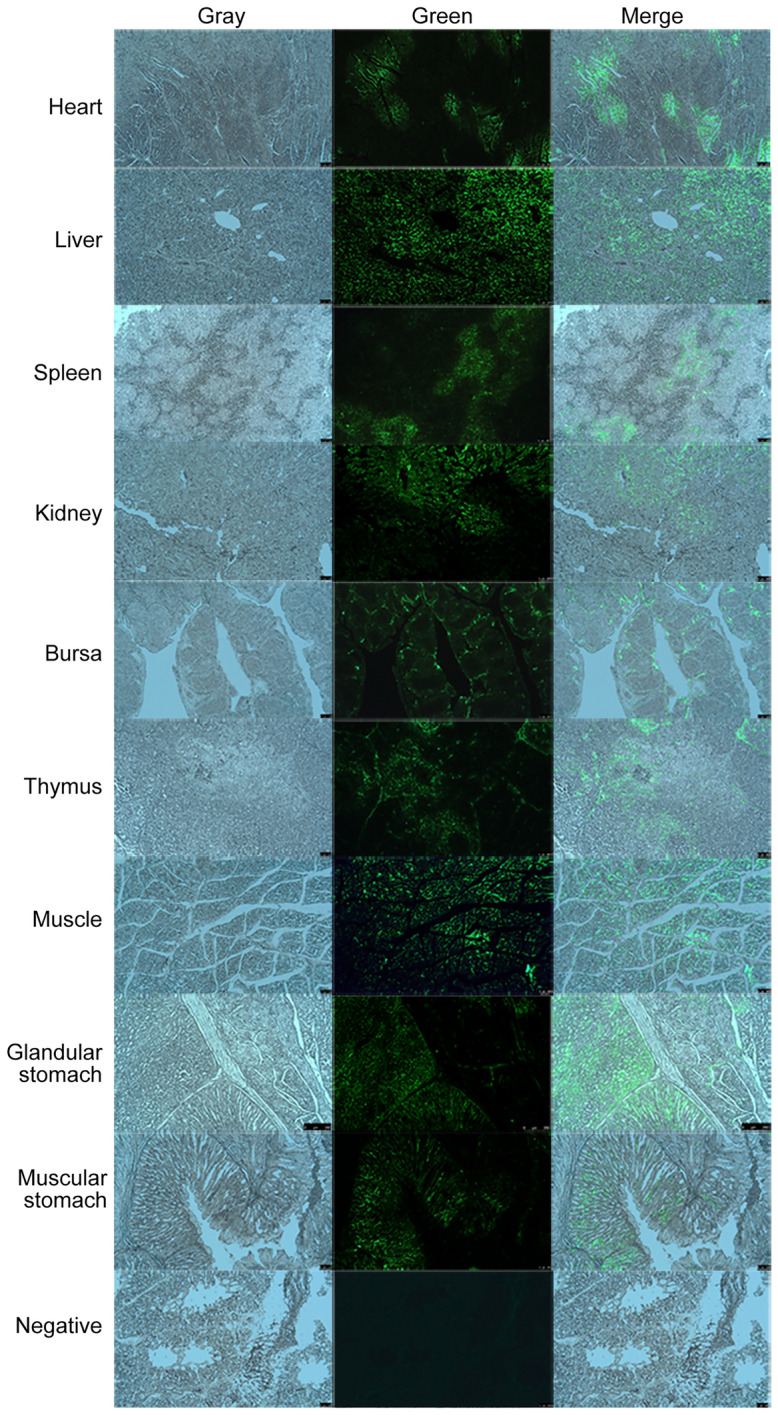
**
Green fluorescence in different chicken tissues from chTERT-overexpressing group was observed in frozen sections (×50).** Since the eukaryotic expression plasmid (pCAGGS-chTERT-eGFP) of chTERT gene and its recombinant lentiviral particle (pLV-chTERT-FLAG + eGFP) both carry eGFP gene, green fluorescence will appear if the exogenous chTERT gene is successfully expressed in chickens, which indicating that the in vivo transfection was successful.

**Figure 3:**
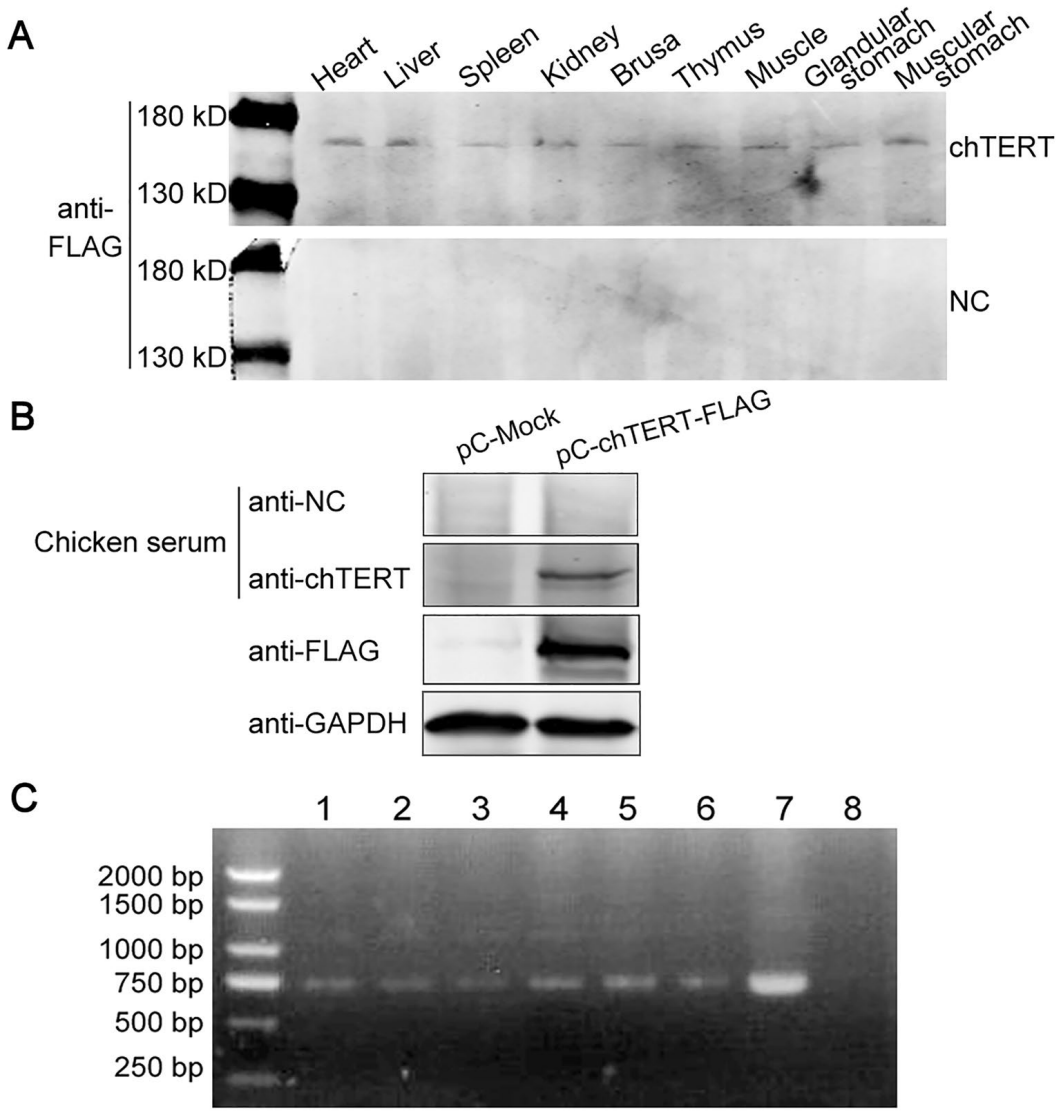
**
chTERT gene was successfully overexpressed in chickens. A** Western blot analysis of the expression of chTERT-FLAG fusion protein in various tissues after transfection in vivo. **B** The chTERT-FLAG protein expressed in 293 T cells in vitro and its control (mock) were used as antigens, and chicken serum was used as an antibody for Western blot analysis. The results showed that when chicken serum overexpressing chTERT was used as an antibody, a band of approximately 150 kD could be successfully detected, which was consistent with the positive control (FLAG antibody) but not detected in the control group, indicating that chTERT was successfully overexpressed in vivo. **C** Chicken blood cell DNA was extracted, and the eGFP gene was amplified by PCR. Lanes 1–3: chicken blood DNA of the chTERT overexpression group; lanes 4–6: chicken blood DNA in the control group; lane 7: positive control; lane 8: negative control. The results showed that the eGFP gene with a size of approximately 720 bp could be successfully amplified from chicken blood DNA, indicating that the in vivo transfection was successful. Since chTERT and eGFP genes were fused and overexpressed in the experimental group, and eGFP gene was only carried in the control group, eGFP gene could also be amplified in the blood DNA even in the control group without overexpression of chTERT.

Before in vivo transfection, chTERT protein expression was verified at the cellular level in vitro. The data showed that chTERT protein was successfully expressed in vitro; the plasmids and lentivirus could be used for in vivo transfection of chickens (Additional files 1 and 2). After transfection in vivo, frozen sections of tissue viscera of a chicken from chTERT-overexpressing group were randomly collected, and the results showed that green fluorescence was observed (Figure 2). In addition, total proteins from different tissues and organs were extracted, and the FLAG Monoclonal antibody was used as a primary antibody for Western blot analysis. The results showed that the chTERT-FLAG fusion protein could be successfully detected in the chTERT-overexpressing chicken group (Figure 3A). Moreover, Western blot analysis was also performed using the chTERT-FLAG protein expressed in 293 T cells in vitro as an antigen, and chicken serum of the chTERT-overexpressing group as the antibody. The results showed that a band of approximately 150 kD was successfully detected, consistent with the results of the positive control (FLAG antibody), and the control group did not show this band (Figure 3B). Finally, chicken blood cells were collected, DNA was extracted, and the eGFP gene in the extracted DNA was amplified by PCR. The results showed that the eGFP gene with a size of 720 bp could be successfully amplified in the blood DNA of the chickens infected with lentivirus and injected with the plasmid (Figure 3C), which further confirmed that the exogenous chTERT gene was successfully overexpressed in chickens.

### Continuous overexpression of chTERT in vivo can significantly promote chicken growth before tumor formation induced by ALV-J

**Figure 4:**
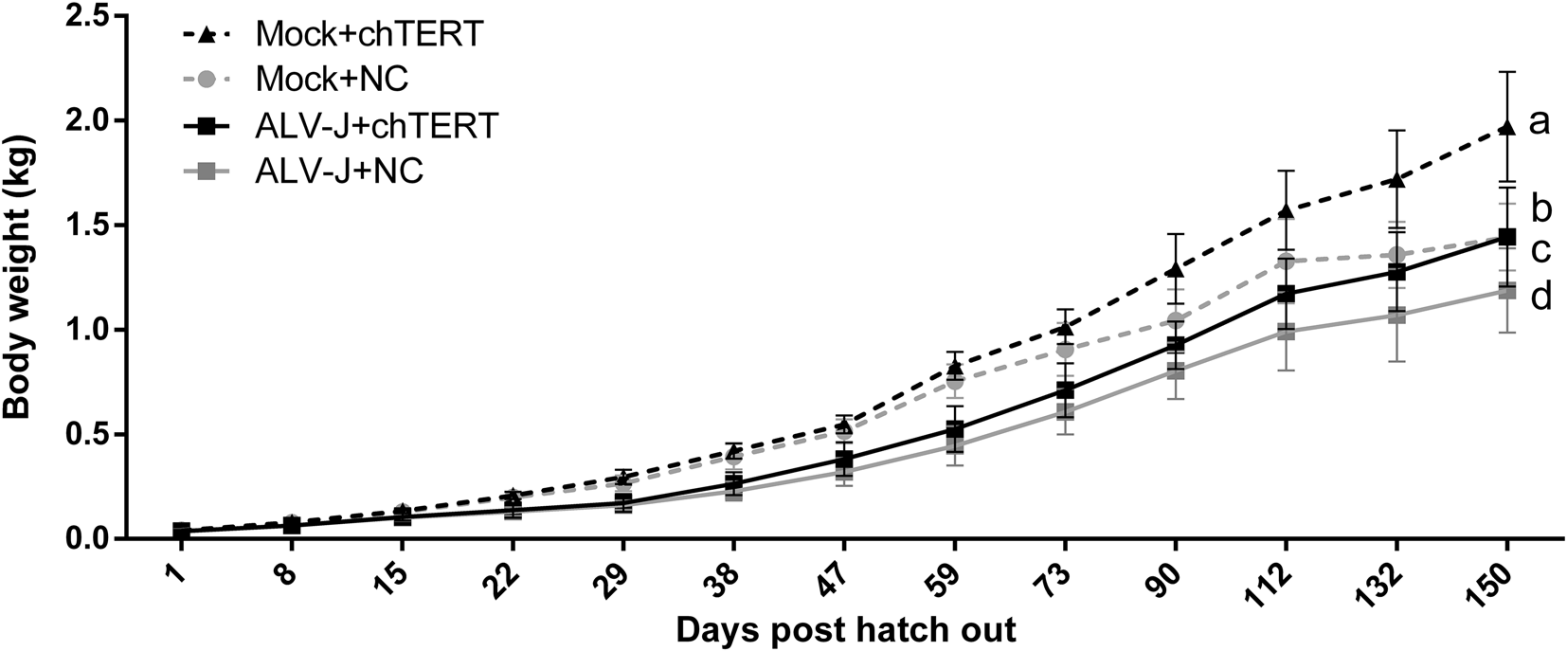
**
Body weight monitoring of chickens in each experimental group.** In vivo overexpression of chTERT significantly promoted chicken growth before the formation of ALV-J tumor, while ALV-J significantly inhibited chicken growth.

During 156 days of feeding, the weight of chickens in the different experimental groups was monitored regularly. It should be noted that, since the diseased chickens and the dead chickens suspected of having tumors will undergo pathological autopsy immediately, all the data of body weight monitoring were from the period before the formation of ALV-J tumor. The results (Figure 4) showed that ALV-J infection could significantly inhibit the weight increase of chickens compared with that of the healthy group. In the ALV-J challenge group or its control group, chTERT overexpression significantly accelerated the weight increase of chickens, and this difference was more significant in the challenge group. These results suggest that chTERT can resist the growth inhibition caused by ALV-J to a certain extent, suggesting that chTERT overexpression can promote the growth of chickens before the formation of ALV-J tumor.

### In vivo overexpression of chTERT enhanced the expression levels of β-catenin and c-Myc in chicken plasma

**Figure 5:**
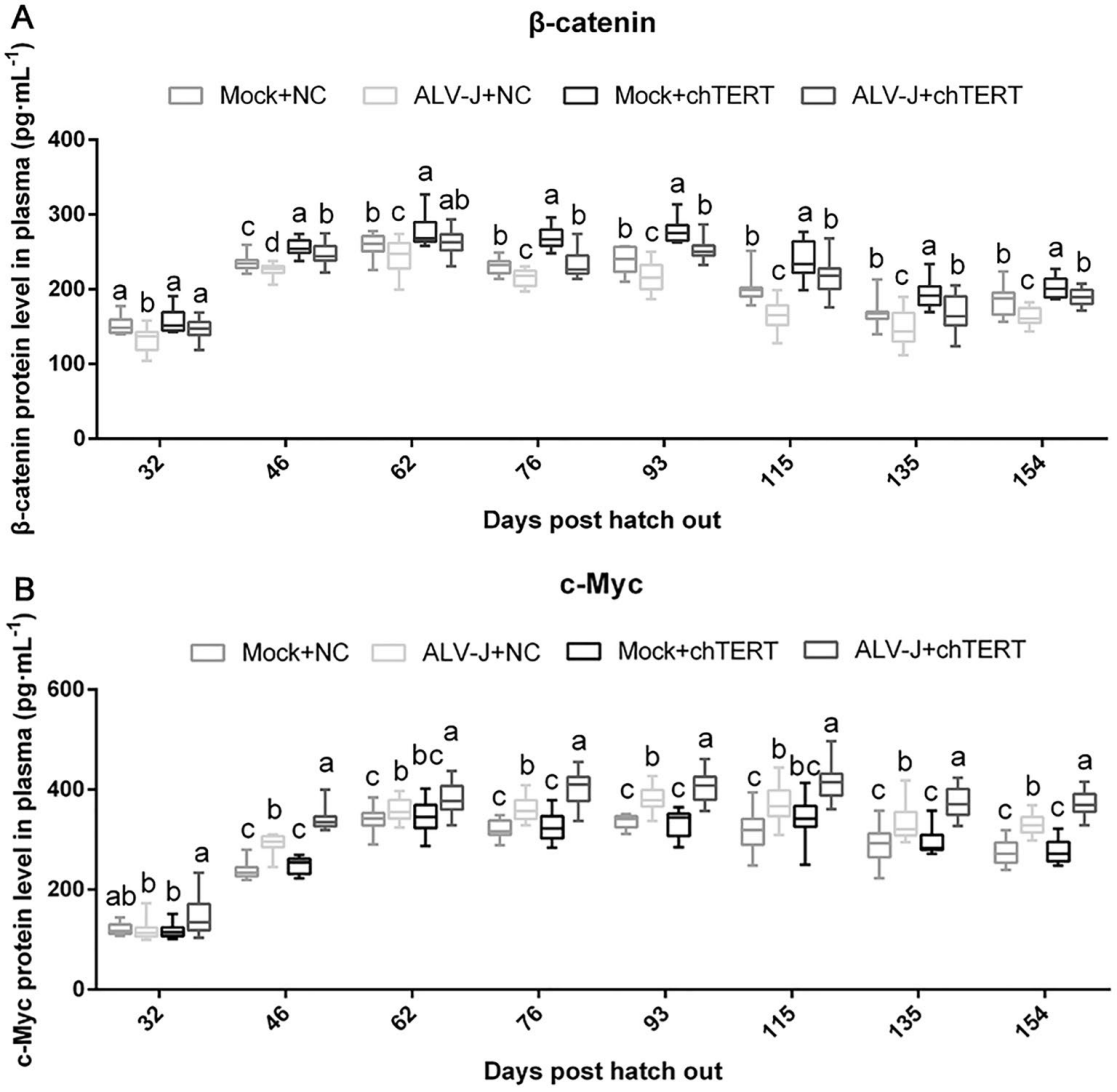
**
Expression of β-catenin (A) and c-Myc (B) in chicken plasma of different experimental groups.** After infection with ALV-J, the protein expression level of β-catenin in plasma was significantly downregulated, but the protein expression level of c-Myc was significantly increased. After chTERT overexpression, the β-catenin protein expression level in plasma was significantly upregulated in both the challenge group and the healthy group, and c-Myc protein expression in plasma was significantly promoted in the challenge group.

The protein expression levels of β-catenin and c-Myc in the plasma of different experimental groups were determined by ELISAs. The results showed that the expression of β-catenin in the plasma of the chickens infected with ALV-J was significantly downregulated, and after overexpression of chTERT gene, the expression level of β-catenin was significantly upregulated (Figure 5A). Combined with Figure 4, the results indicated that chTERT promoted the growth of chickens by enhancing the activity of the β-catenin signaling pathway and compensated for the growth inhibition caused by ALV-J to some extent. However, the expression of c-Myc in plasma increased significantly after ALV-J infection (Figure 5B). In addition, it was found that chTERT mainly played a growth-promoting role in healthy chickens, because there was no significant change in plasma c-Myc expression levels after chTERT overexpression in vivo. However, in the ALV-J-infected group, chTERT showed both growth and cancer promotion. The expression level of c-Myc in plasma was significantly increased after chTERT overexpression in vivo, suggesting that chTERT could further enhance the expression level of c-Myc and thus promote the occurrence of tumors.

### Collection and identification of ALV-J tumors

**Figure 6:**
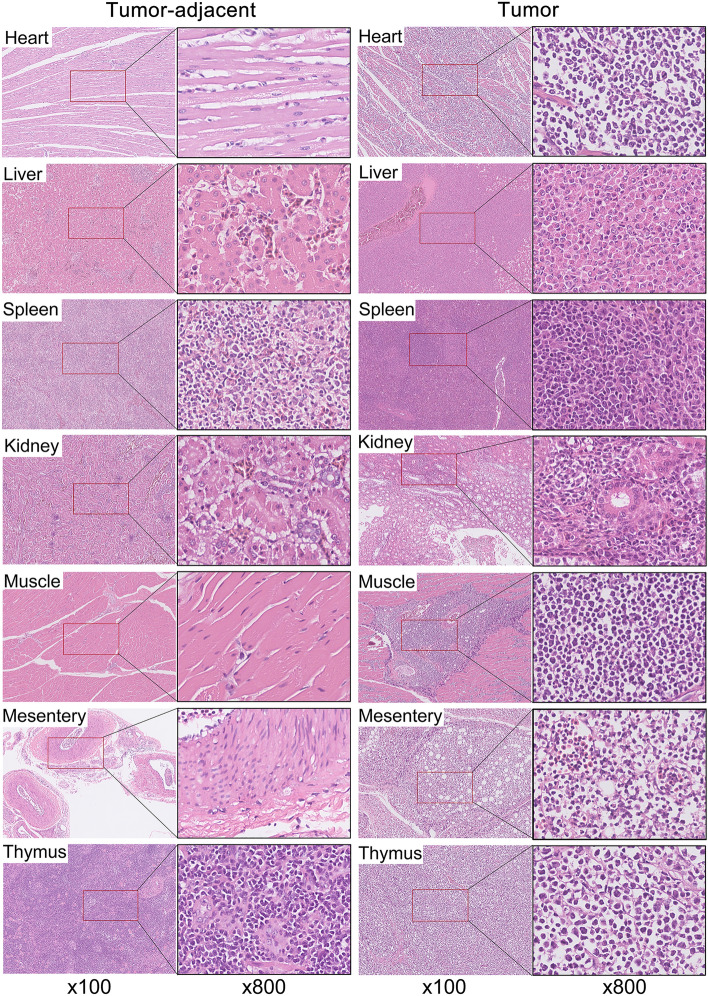
**HE staining of different tumor tissues.**. HE staining of the suspected tumor tissue showed that normal tissue and organ structures were destroyed, and a large number of lymphocytes or myeloid tumor cells infiltrated the tumor tissue. The cells were large and round, with abundant cytoplasm and obvious eosinophilic granules, as well as a small number of infiltrating monocytes and blood cells. Moreover, cells atdifferent stages of differentiation were observed and were round, conical, spindle and long fiber types. The colored particles in some cells had no autolysis. In summary, the suspected tumor tissues collected were confirmed to be tumors caused by chronic transformed ALV-J.

Pathological autopsy was performed on 3 chickens (No. 93, No.139, No. 129) with suspected tumor disease in the chTERT overexpression group, the results showed typical mesenteric sarcoma, grayish white tumor nodules, thymus enlargement, heart tumors, large grayish white tumor nodules in the pectoral muscle, and grayish white tumor nodules in the liver (Additional files 3 A–O). Moreover, in order to obtain more tissue samples for subsequent research, pathological autopsy was performed on chickens with suspected ALV-J tumor diseases from clinical larger scale poultry farms. There were obvious grayish white tumor nodules in the liver, spleen and kidney (Additional files 3P–U). The corresponding tissues of healthy chickens of the same age were collected as normal tissues. All the ALV-J tumor tissues collected from artificial tumorigenic experiment and clinical poultry farms were used as the follow-up study samples, and after isolation and identification of ALV (Additional file 4), HE staining (Figure 6), whole genome amplification of ALV-J (Additional file 5) and sequencing analysis (Additional file 6), a total of 25 samples of tumor tissues induced by chronic transformed ALV-J were collected (Additional file 7) [26].

### chTERT may play an important role in tumor formation induced by ALV-J and chTERT was highly expressed in tumors

**Figure 7:**
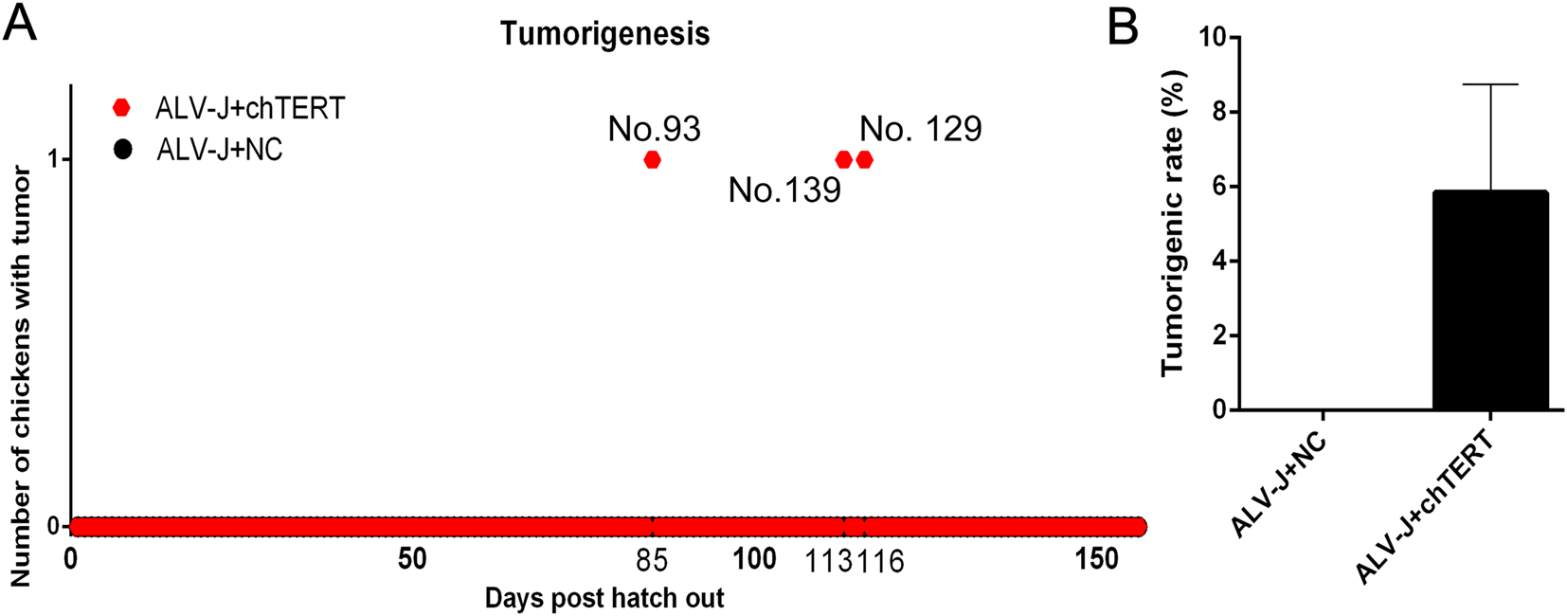
**Continuous overexpression of chTERT in chickens promoted ALV-J induced tumor formation.** **A** The number of chickens with tumors and the timing of its onset; **B** Tumorigenic rate of ALV-J.

**Figure 8:**
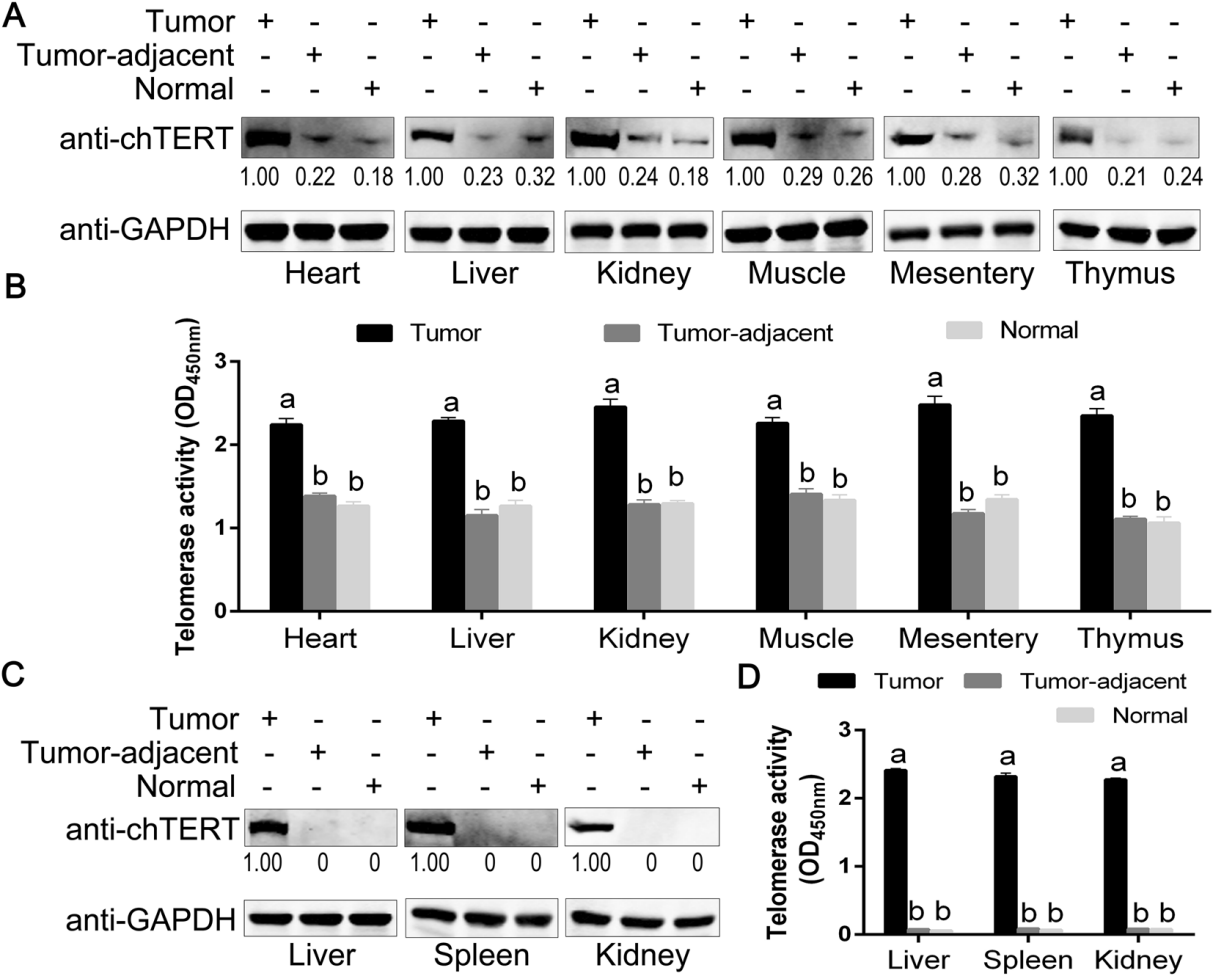
**
chTERT was significantly highly expressed in the tumor tissues induced by ALV-J.** Analysis of chTERT protein expression levels (**A**) and telomerase activity (**B**) in different tumor tissues, tumor-adjacent tissues and corresponding normal tissues obtained by artificial tumor-inducing experiments. Analysis of the chTERT protein expression levels (**C**) and telomerase activity (**D**) in different tumor tissues, tumor-adjacent tissues and corresponding normal tissues obtained from clinical cases.

At the end of 156-day artificial tumor-inducing experiment, pathological autopsy was performed on all surviving chickens. The results (Figure 7A) showed that only the above 3 chickens (No. 93 and No.139, No. 129) in the two batches of chTERT-overexpression group (*n* = 26*2) developed 7 solid tumors, the tumorigenic rates were 3.8% (1/26) and 7.7% (2/26), respectively. While no tumors occurred in the control group (*n* = 26*2), suggesting that overexpression of chTERT gene in vivo may promote tumor formation induced by ALV-J (Figure 7B). Since the tumorigenic rates of ALV-J in the two batches of chTERT-overexpression group in this study were low and significantly difference, more sufficient data to further support this conclusion would be better. Proteins were extracted from tumors, tumor-adjacent and normal tissues in ALV-J artificial tumor-inducing experiment, and the expression of chTERT and telomerase activity were detected. The results showed that the expression level of chTERT and telomerase activity were significantly higher in all ALV-J tumor tissues than that in the tumor-adjacent and normal tissues (Figure 8A and B). Since the tumor tissues obtained by the artificial tumor-inducing experiment were all from the chTERT overexpression group, there were low levels of chTERT expression and positive telomerase activity in the corresponding tumor-adjacent tissues and normal tissues. However, there was no chTERT expression in the tumor-adjacent and normal tissues in the clinical samples obtained from poultry farms, and telomerase activity was negative (Figure 8C and D). Overall, chTERT protein and telomerase activity always showed a trend of significantly higher expression in ALV-J tumor tissues than that in tumor-adjacent and normal tissues.

### The high expression level of chTERT in ALV-J tumors enhanced the activity of the Wnt/β-catenin signaling pathway and the expression level of c-Myc

**Figure 9:**
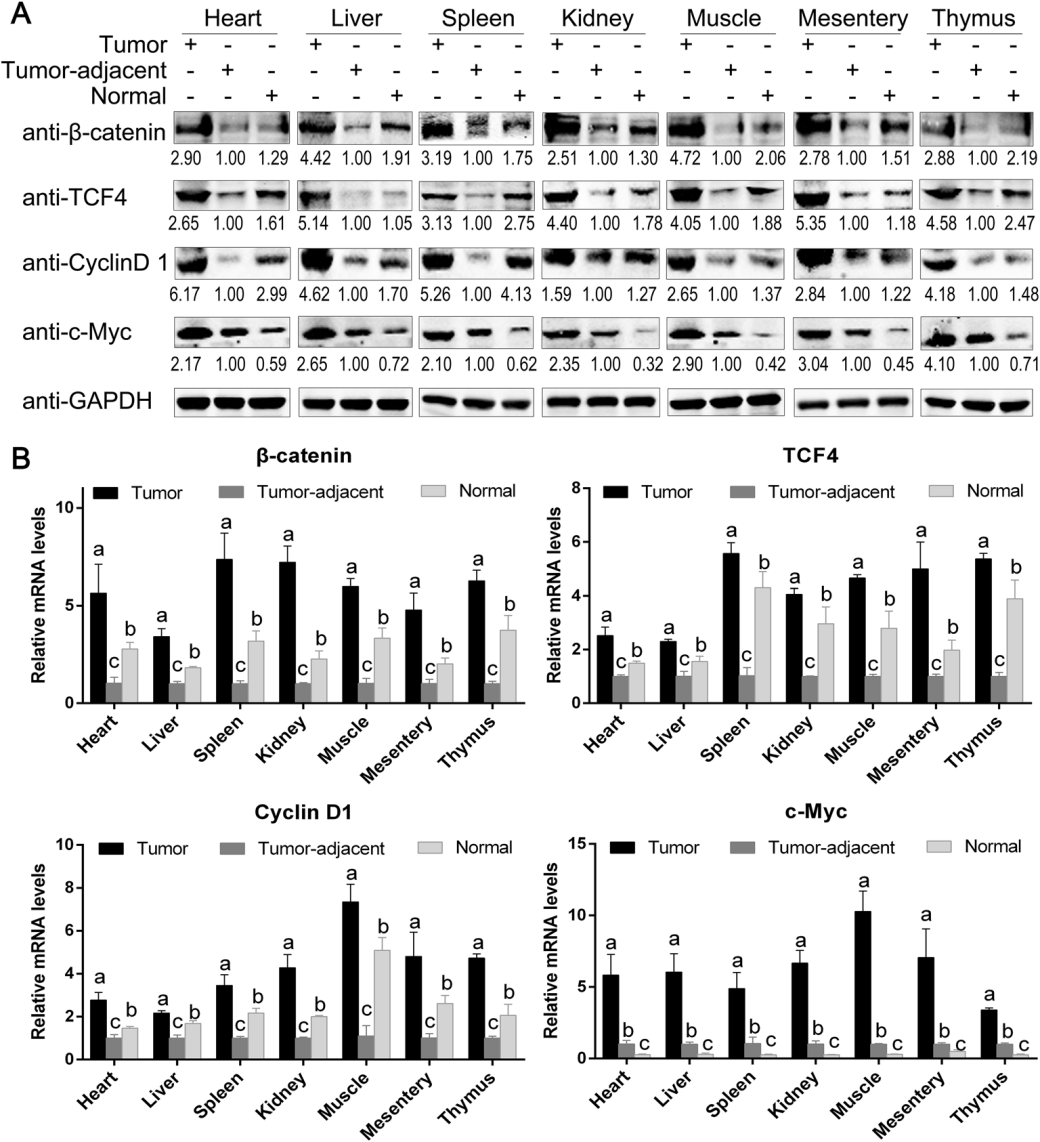
**
The high expression of chTERT in ALV-J tumors enhanced the activity of Wnt/β-catenin signaling pathway.** Protein (**A**) and mRNA (**B**) expression levels of β-catenin, TCF4, Cyclin D1 and c-Myc in different tumor tissues, tumor-adjacent tissues induced by ALV-J and corresponding normal tissues.

**Figure 10:**
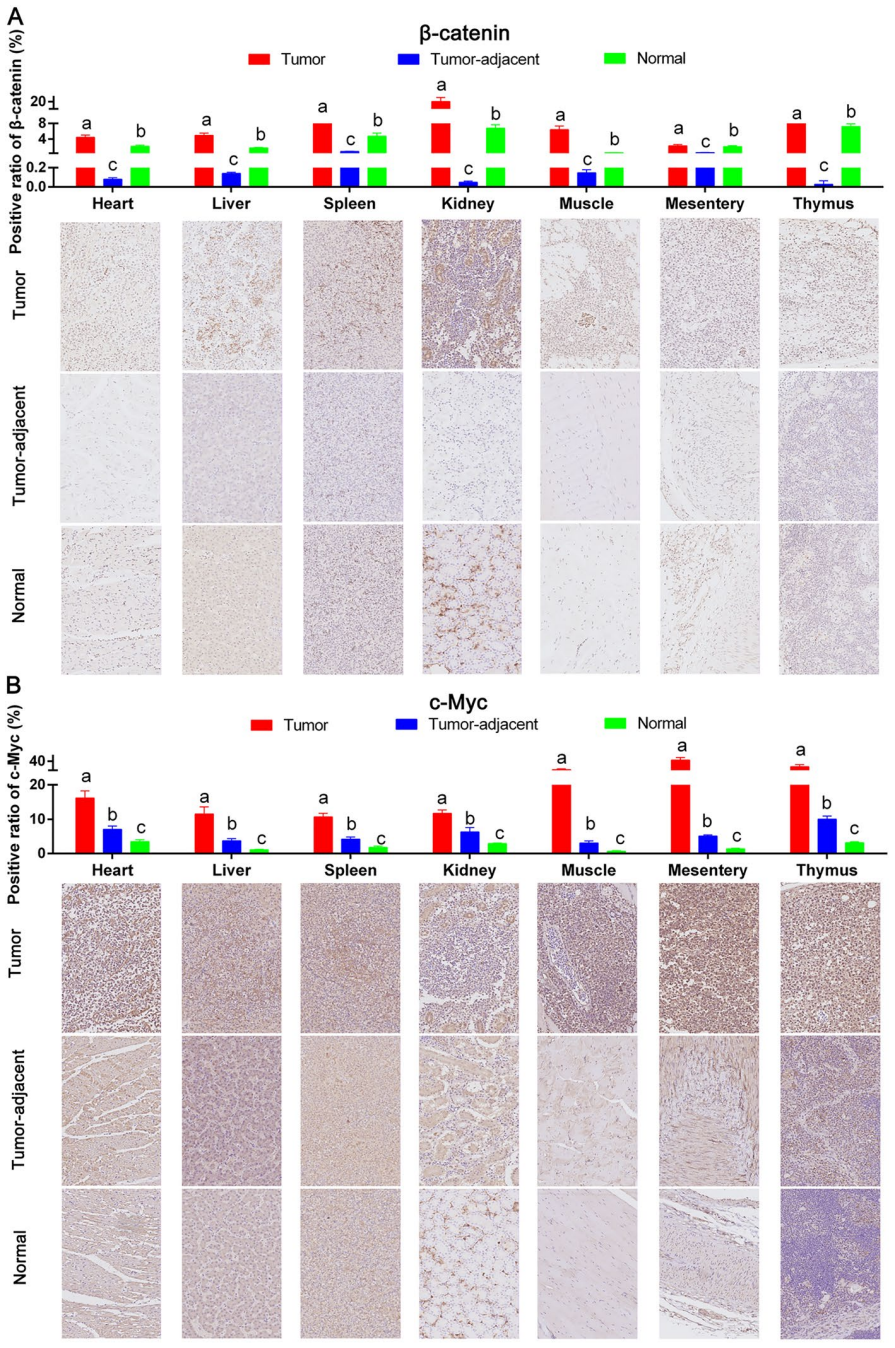
**The high expression of chTERT in ALV-J tumors promoted the expression of β-catenin and c-Myc.** Immunohistochemical analysis of the expression levels of β-catenin (**A**) and c-Myc (**B**) in different tumor tissues, tumor-adjacent tissues and normal tissues (×400).

Protein and mRNA were extracted from ALV-J tumors, tumor-adjacent and normal tissues, and the correlation between the high expression of chTERT in tumors and the activity of the Wnt/β-catenin signaling pathway was analyzed by Western blot and RT-qPCR. The results (Figure 9A) showed that the protein expression levels of β-catenin, TCF4 and cyclin D1 in the tumor-adjacent tissues were significantly lower than those in the normal tissues, suggesting that ALV-J inhibited cell growth and development by decreasing the Wnt/β-catenin signaling pathway. The expression level of the proto-oncogene c-Myc in the tumor-adjacent tissues was significantly higher than that in the normal tissues. In contrast, the protein expression levels of β-catenin, TCF4, Cyclin D1 and c-Myc in the ALV-J tumors were significantly higher than those in the tumor-adjacent and normal tissues, suggesting that the high expression of chTERT in tumors was positively correlated with the activity of the Wnt/β-catenin signaling pathway. In other words, chTERT not only promoted the expression level of c-Myc induced by ALV-J but also promoted the growth of tumor cells. The data obtained by RT-qPCR (Figure 9B) and immunohistochemistry (Figure 10) were also consistent with the above conclusions.

### The proportion of apoptotic cells in ALV-J tumors was significantly lower than that in tumor-adjacent tissues

**Figure 11:**
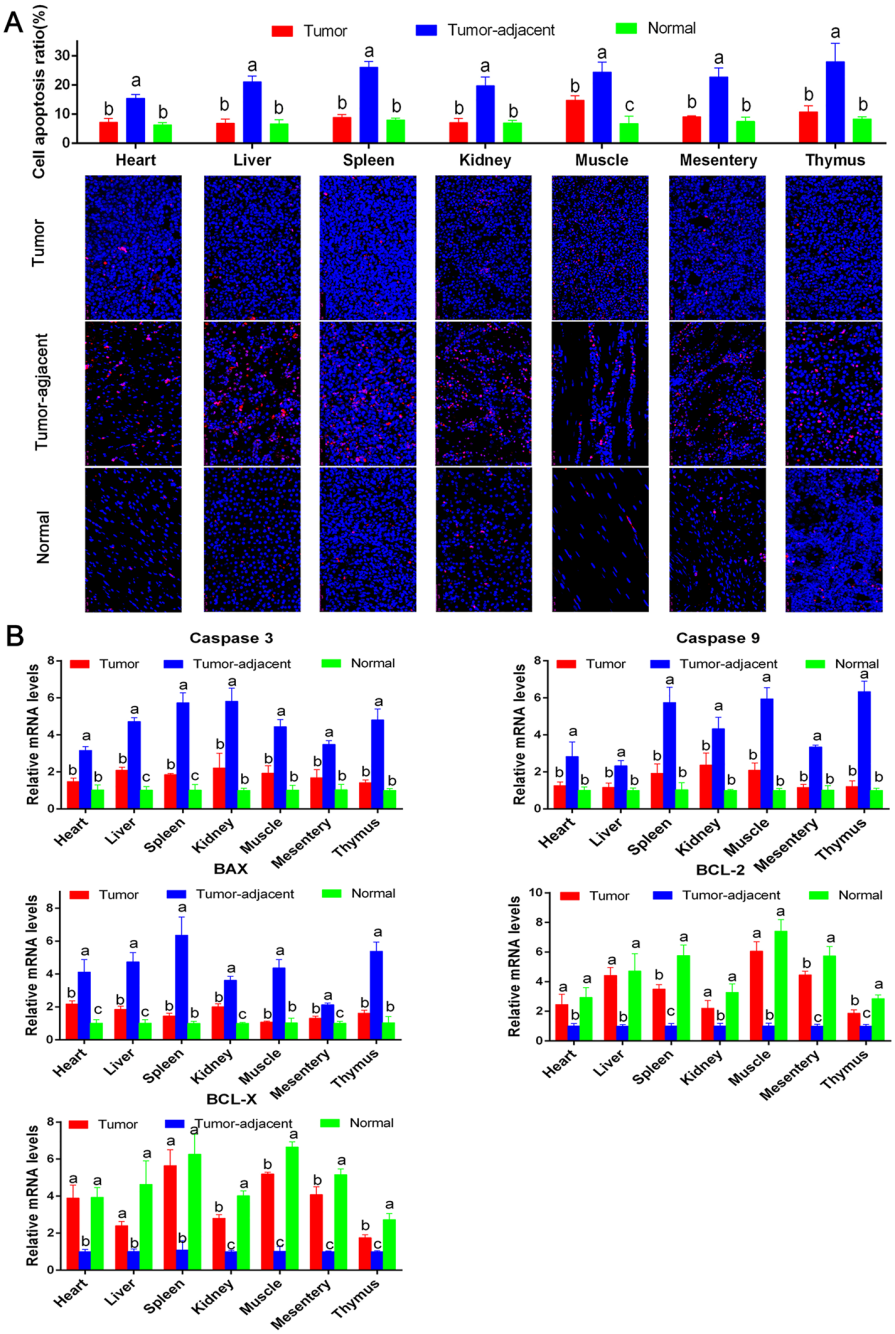
**
High expression levels of chTERT in ALV-J tumors inhibited cell apoptosis. A** TUENL staining was used to analyze the proportion of apoptotic cells in different tumor tissues, tumor-adjacent tissues and normal tissues (×400). **B** The mRNA expression levels of Caspase 3, Caspase 9, BAX, BCL-X and BCL-2 in different tumor tissues, tumor-adjacent tissues and normal tissues.

TUNEL detection was performed on ALV-J tumors, tumor-adjacent and normal tissues to analyze the apoptosis of host cells. The results (Figure 11A) showed that when ALV-J infected chickens, the proportion of apoptosis in the tumor-adjacent tissues was significantly higher than that in the normal tissues. The percentage of apoptosis in tumors was significantly lower than that in the tumor-adjacent tissues. The reason for this result was presumed to be due to the significantly high expression of chTERT in tumors, which may play an important role in inhibiting cell apoptosis, compensating for the apoptosis induced by ALV-J to some extent and thus promoting the transformation of normal cells into cancer cells. However, more direct experiments data are needed to verify that. The RT-qPCR results (Figure 11B) showed that the mRNA levels of the proapoptotic genes Caspase 3, Caspase 9 and BAX in the tumor-adjacent tissues were significantly higher than those in the normal tissues, while the levels of the antiapoptotic genes BCL-X and BCL-2 in the tumor-adjacent tissues were significantly lower than those in the normal tissues. The mRNA levels of proapoptotic genes in the tumors were significantly lower than those in the tumor-adjacent tissues, and the levels of the antiapoptotic genes in the tumors were significantly higher than those in the tumor-adjacent tissues.

### The high expression level of chTERT in tumors upregulated the replication level of ALV-J

**Figure 12:**
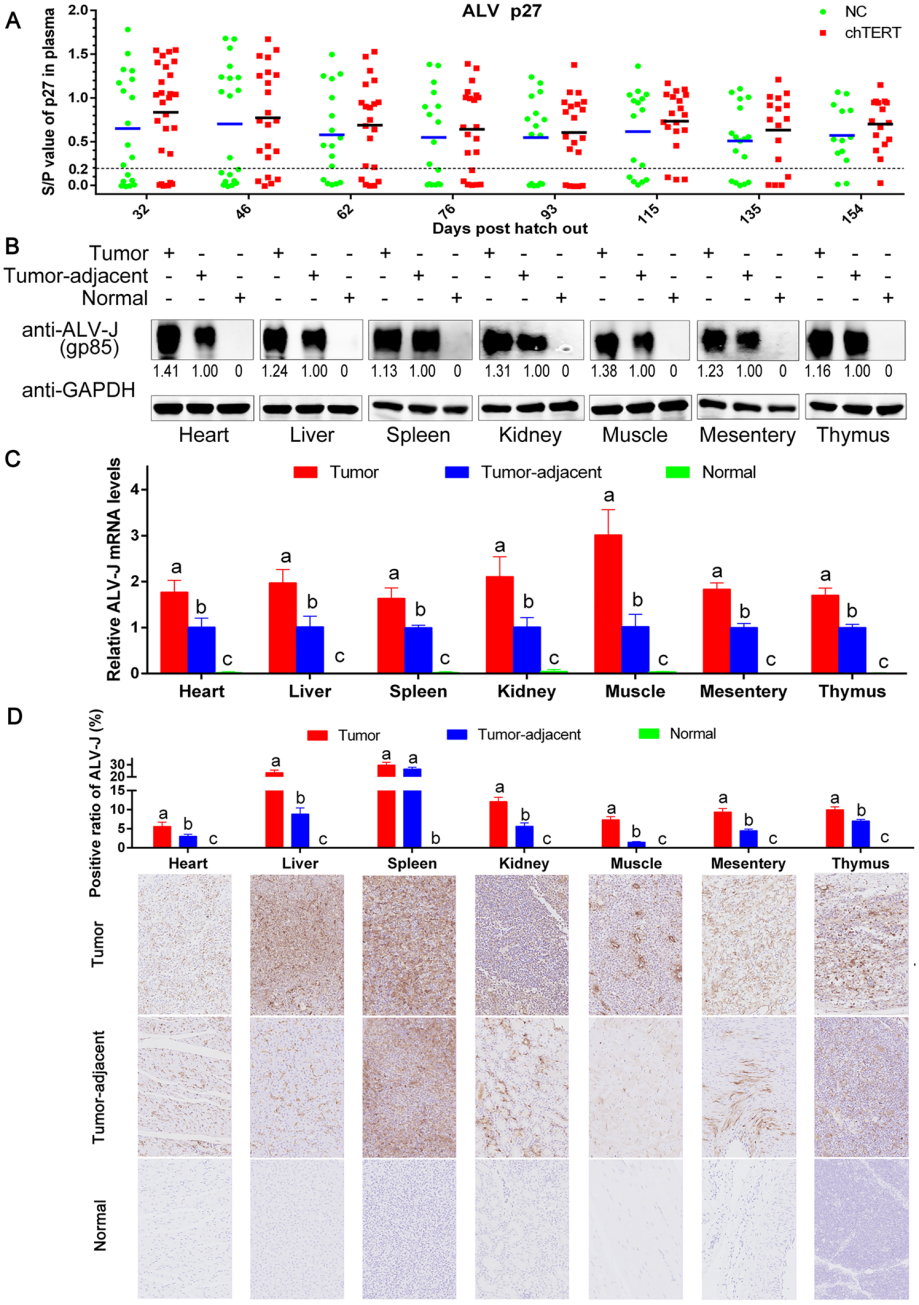
**High expression levels of chTERT in tumors promoted the replication of ALV-J. A** In the plasma of chickens overexpressing chTERT, the p27 antigen content was slightly higher than that in the control group, but there was no significant difference. Protein (**B**) and mRNA (**C**) expression levels of ALV-J in different tumor tissues, tumor-adjacent tissues and normal tissues. **D** Immunohistochemical analysis of ALV-J replication levels in tumor tissues, tumor-adjacent tissues and normal tissues (×400).

Our previous study suggested that chTERT could promote the replication of chronic translational ALV-J in LMH cells. To further verify this conclusion, we detected the content of ALV p27 antigen in chicken plasma of different experimental groups by ELISA. The results showed that the expression level of plasma p27 antigen increased after chTERT overexpression overall, but there was no significant difference (Figure 12A). Then, the replication level of ALV-J in tumors and tumor-adjacent tissues was compared by Western blot (Figure 12B) and RT-qPCR (Figure 12C). The results showed that the protein level and mRNA level of ALV-J gp85 in the tumors were significantly higher than those in the tumor-adjacent tissues, suggesting that the high expression of chTERT in tumors promoted the replication of chronic translational ALV-J. The results of ALV-J immunohistochemistry also confirmed this conclusion (Figure 12D). It should be noted that AL is a viral tumor disease caused by ALV, so it is reasonable that the virus also exists in the tumor-adjacent tissues [27, 28].

## Discussion

Our previous study suggested that chTERT and Wnt/β-catenin signaling pathway in LMH cells can regulate each other to inhibit cell apoptosis and promote the replication of ALV-J [14]. To verify this conclusion and directly determine the role of chTERT in ALV-J tumor inducing, we conducted an artificial tumor-inducing experiment, starting from the host itself, through in vivo plasmid injection and lentivirus infection to overexpress chTERT in chickens continuously to analyze its role in the tumorigenicity of ALV-J. On this basis, chicken plasma, tumors, and tumor-adjacent and normal tissues were used to analyze the effect of high expression of chTERT in tumors on the Wnt/β-catenin signaling pathway, host cell apoptosis and ALV-J replication.

It should be noted that due to the limitations of experimental conditions and technical means, this study could not accurately find the time point at which ALV-J tumors first appeared in each chicken body, and thus could not evaluate the impact of chTERT overexpression on tumor occurrence and development after tumor emergence. In this study, pathological autopsy was performed only on chickens with clinical symptoms to determine whether or not tumors appeared, and it is not known when tumors appeared. Therefore, this study cannot accurately analyze the correlation between the influence of chTERT on the occurrence and development of ALV-J tumor and the change of chicken body weight. If experimental conditions and technical means allow, we speculate that chTERT will inevitably inhibit the growth of chickens while exacerbating the occurrence and development of tumors. During 156 days of chronic transformed ALV-J artificial tumorigenic experiment, only 3 chickens developed solid tumors. When a tumor was suspected, the chicken was immediately killed and pathologically dissected. Naturally, the chickens with tumors were not subject to follow-up weight monitoring. In other words, artificial tumorigenic experiment for 156 days, chickens with weight monitoring data at this time can be regarded as in the period before the formation of ALV-J tumor, which is also the default specific period in the conclusion description in this chapter. From the perspective of population, the overexpression of chTERT in vivo can promote the increase of body weight and growth of experimental chickens during the buffer period.

ELISAs results showed that the expression level of β-catenin in chicken plasma was significantly decreased after ALV-J infection, suggesting that ALV-J decreased the activity of the Wnt/β-catenin signaling pathway and inhibited the growth and development of chickens, which was consistent with previous research [29]. Moreover, overexpression of chTERT significantly upregulated the expression level of β-catenin to increase body weight and promote growth [30]. In contrast, ALV-J infection resulted in a significant increase in the expression of the proto-oncogene c-Myc, reflecting the carcinogenic property of ALV-J itself [31]. In healthy chickens, the expression level of c-Myc gene was not significantly affected even after chTERT overexpression because there was no stimulation of ALV-J. In the challenge group, on the basis of the carcinogenic effect of ALV-J itself, the expression of c-Myc induced by ALV-J was significantly increased after overexpression of chTERT, reflecting the potential carcinogenic effect of chTERT [1, 22].

In this study, after 156 days of feeding, only 3 chickens were found to develop 7 solid tumors induced by the ALV-J Hc1 strain, and the tumor-inducing rate was much lower than expected. As reported by Pandiri et al., inoculated 10 000 TCID_50_ of Hc1 virus through the allantoic cavity on the 5th day of chicken embryos, the tumorigenicity rate was 100% within 224 days, and the average time of tumor induction was 180 days [4]. In this study, 10 000 TCID_50_ of Hc1 virus was inoculated through the allantoic cavity on the 11th day in chicken embryos, which were housed for only 156 days. Considering the time of virus inoculation and the housing period, the selected time point of virus inoculation and feeding period in this study could not achieve the tumorigenic effect of Hc1 in the study of Pandiri et al. [4]. Given the differences in chicken strains, it was not surprising that Hc1 had such a low tumorigenic rate in this study. Due to the small number of tumor tissues obtained by artificial tumorigenic experiments, 18 clinical ALV-J tumor tissue samples in breeding poultry farms were collected for follow-up research. On this basis, the expression level of chTERT in all the obtained tumors and their corresponding tumor-adjacent and normal tissues was compared in this study. The results showed that the expression level of chTERT gene and telomerase activity in the tumors were significantly higher than those in the tumor-adjacent and normal tissues, which was consistent with the results of previous studies [1, 22, 32].

Different from a variety of human non-viral cancer diseases such as liver cancer, lung cancer, stomach cancer and thyroid cancer, avian leukosis is a viral tumor disease, and ALV itself plays a crucial role in the process of tumor induction. Without the involvement of ALV itself, it is difficult to induce tumor formation even if chTERT expression level and Wnt/β-catenin signaling pathway activity are significantly increased. Therefore, the replication level of ALV is closely related to the occurrence and development of avian leukosis associated tumors, which is also different from the pathogenesis of most human tumor diseases. Naturally, it was necessary to explore the effect of chTERT on ALV-J replication. To verify the conclusion that chTERT could upregulate the replication level of chronic translational ALV-J, we determined the expression of ALV p27 antigen in the plasma of chickens of the different experimental groups by ELISAs. The results showed that the expression level of p27 in the plasma of the chTERT-overexpressing chickens was slightly higher than that of the control group, but there was no significant difference. This finding was due to the individual differences that existed in each group, and the most important is that the dynamics of viremia in chickens infected with ALV are not constant but dynamic, without a specific pattern [33]. Therefore, the plasma p27 content may be different at different time points, which is also a key and difficult issue in the purification process of avian leukosis. Although there was no significant difference in the antigen content of ALV-J p27 in chicken plasma between the two experimental groups, but from the consideration of a group perspective, chTERT still upregulated the expression level of ALV-J p27 antigen in chicken plasma. Therefore, to corroborate this conclusion, we analyzed the replication levels of ALV-J in its tumors and tumor-adjacent tissues by RT-qPCR, Western blotting and immunohistochemistry. The results showed that the replication level of ALV-J in the tumors was significantly higher than that in the tumor-adjacent tissues, suggesting that the high expression of chTERT in the tumor tissues promoted the replication of ALV-J, which was also regulated by the Wnt/β-catenin signaling pathway [14, 34]; moreover, the replication of ALV-J can upregulate the expression level of c-Myc, so chTERT can also promote the expression of c-Myc and then promote the occurrence and development of tumors.

**Figure 13:**
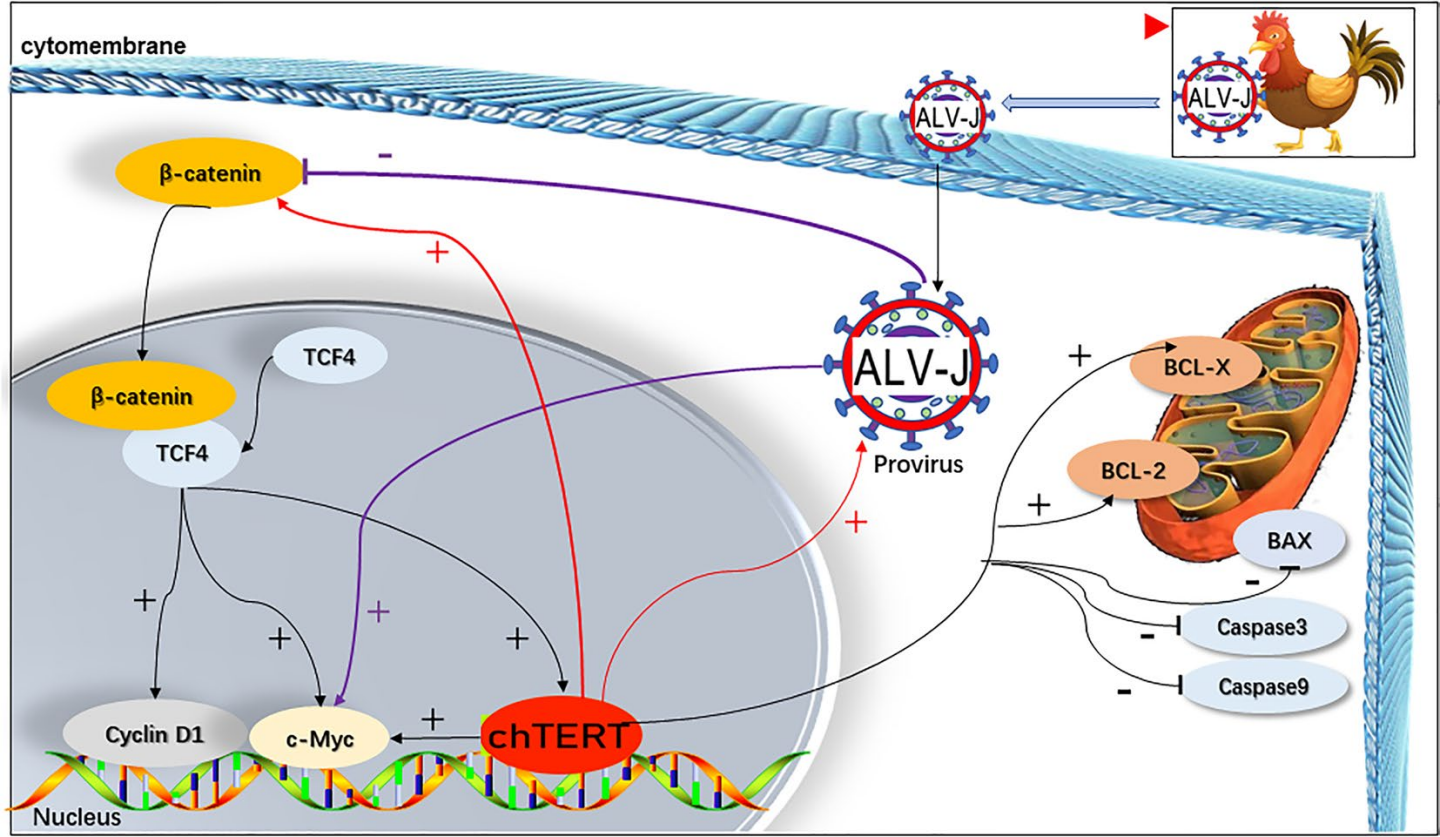
**
Schematic diagram of the molecular mechanism by which chTERT promotes ALV-J to induce tumor formation.** In this study, continuous overexpression of chTERT gene in chickens can promote the tumorigenicity of chronic translational ALV-J by enhancing the Wnt/β-catenin signaling pathway and further increased the expression level of c-Myc, inhibited tumor cell apoptosis, and promoted the replication of ALV-J.

In summary, continuous overexpression of chTERT gene in chickens can promote the tumorigenicity of chronic translational ALV-J by enhancing the Wnt/β-catenin signaling pathway and further increased the expression level of c-Myc, inhibited tumor cell apoptosis, and promoted the replication of ALV-J (Figure 13). In this study, the role and molecular mechanism of chTERT in chronic transformed ALV-J tumorigenicity were preliminarily analyzed, which will contribute to further elucidating the mechanism of ALV tumorigenicity.

## Supplementary Information

**Additional file 1.** **chTERT protein was successfully expressed in in vitro cells**. Observation of LMH cells after transient transfection (A) and 293 T cells after packaging lentivirus, (B) under a fluorescence microscope (×50)

**Additional file 2. Western blot analysis of chTERT expression in vitro**. (A) chTERT eukaryotic expression plasmid was transfected into LMH cells. (B) Packaging of recombinant lentivirus particles of chTERT in 293 T cells

**Additional file 3.** **Pathological autopsy of sick chickens in artificial tumorigenic experiments (A-O) and clinical cases from breeding poultry farms (P-U)**. During the housing period of the artificial tumorigenic experiment, 3 chickens developed solid tumors (No. 93 (A-F), No. 139 (G-L) and No. 129 (J-O)) at 85 days, 113 days, and 116 days after hatching, respectively. Pathological autopsy revealed typical mesenteric sarcoma (B, C, J), dissection of the kidney revealed grayish white tumor nodules (D, E), thymus enlargement (K), cardiac tumors **(I)**, large grayish white tumor nodules in the pectoral muscle (L) and grayish white tumor nodules in the liver (M, N, O). The livers and spleens of sick chickens I and II were enlarged without tumor nodules (F, H). Moreover, the necropsy of chickens with suspected tumors from clinical farms (P-U) showed prominent grayish white tumor nodules in the liver (P, Q), spleen (S, T, U) and kidney (R). The typical grayish white nodules are indicated by black arrows

**Additional file 4.** **Isolation and identification of ALV-J**. (A) Agarose gel electrophoresis image of PCR products; The results showed that only ALV-J was detected in tumor tissues. lanes 1–22: tumor samples, lane 23: positive control, lane 24: the negative control; (B) The results of RT-qPCR showed that only ALV-J was detected in tumor tissues. (C) Immunohistochemical analysis of ALV-J in tumor tissue (×400); The brown areas are cells that are positive for the target protein (ALV-J gp85). (D) Immunofluorescence staining of ALV-J (×400). ALV-A: avian leukosis virus subgroup A; ALV-B: avian leukosis virus subgroup B; ALV-J: avian leukosis virus subgroup J; ALV-K: avian leukosis virus subgroup K; MDV: Marek’s disease virus; REV: Reticuloendotheliosis virus

**Additional file 5.** **Agarose gel electrophoresis of the amplification products of the ALV-J genome**. The complete genome of chronic transformed ALV-J, including *gag*, *pol*, *env* and *UTR*, was successfully amplified

**Additional file 6.** **Genetic evolution analysis of the whole genome (5’- *UTR-gag-pol-env-UTR*-3’) of ALV-J isolates**. “▲” represents the chronically transformed ALV-J strains which were isolated from breeding poultry farms in this study. The results showed that the ALV-J strains isolated from breeding poultry farm were closely related to other classical chronic transformation ALV-J, such as NX0101 and SCAU-HN06.

**Additional file 7.** **ALV-J tumor tissues and their sources and quantities.**

## Abbreviations

TERT: telomerase reverse transcriptase
chTERT: chicken telomerase reverse transcriptase
ALV-J: avian leukosis virus subgroup J
TRAP: telomeric repeat amplification protocol
ELISA: enzyme-linked immunosorbent assay
TUNEL: terminal-deoxynucleotidyl transferase-mediated nick end labeling
TCID_50_: 50% tissue culture infection dose
RT-qPCR: real-time quantitative PCR
PBS: phosphate-buffered saline
