# Supplementary figures and images for: Chicken telomerase reverse transcriptase promotes the tumorigenicity of avian leukosis virus subgroup J by regulating the Wnt/β-catenin signaling pathway
Source: Vet Res. 2022 Dec 2;53:100. doi: 10.1186/s13567-022-01120-2 (PMC9717515; doi:10.1186/s13567-022-01120-2)

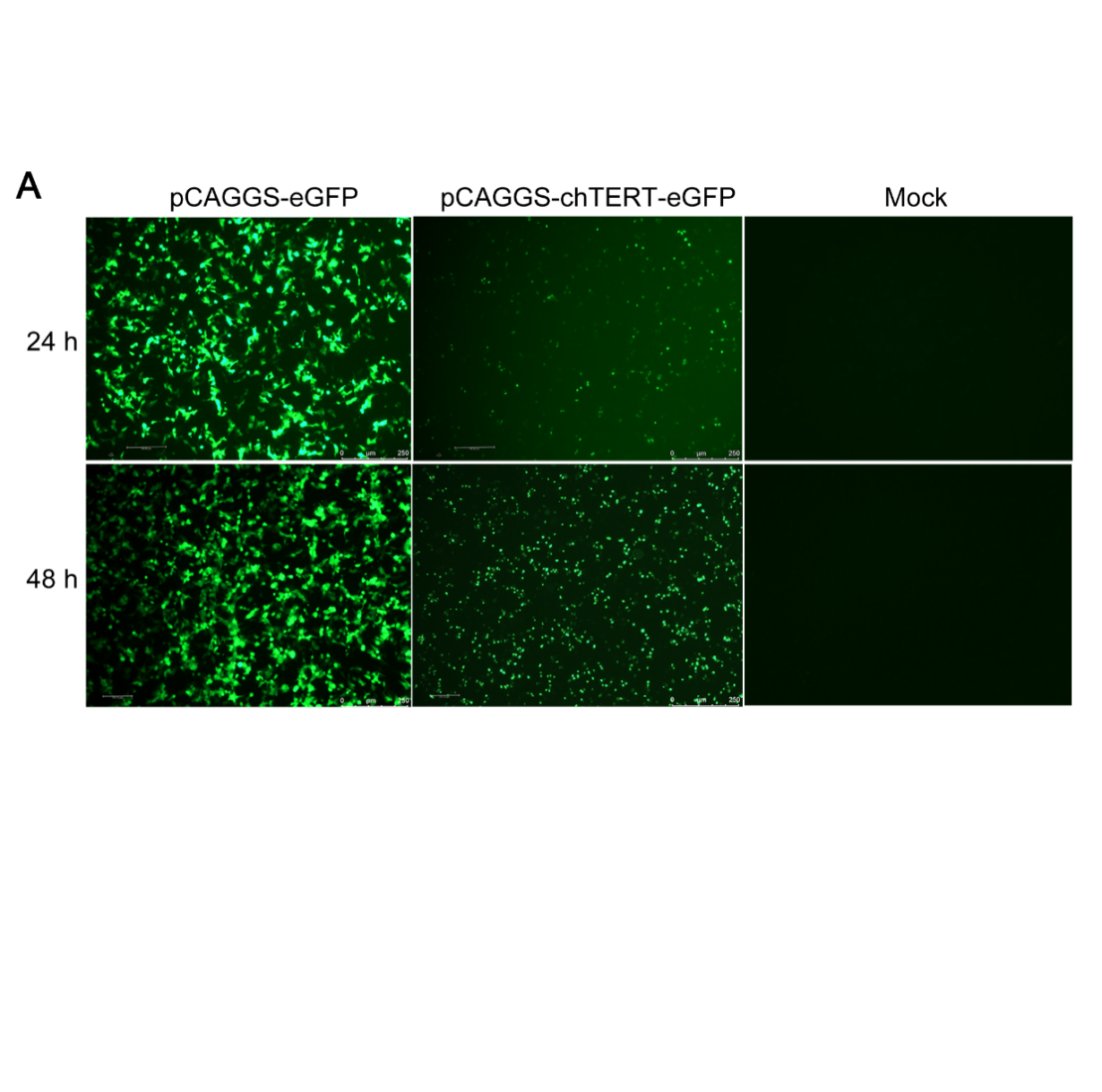


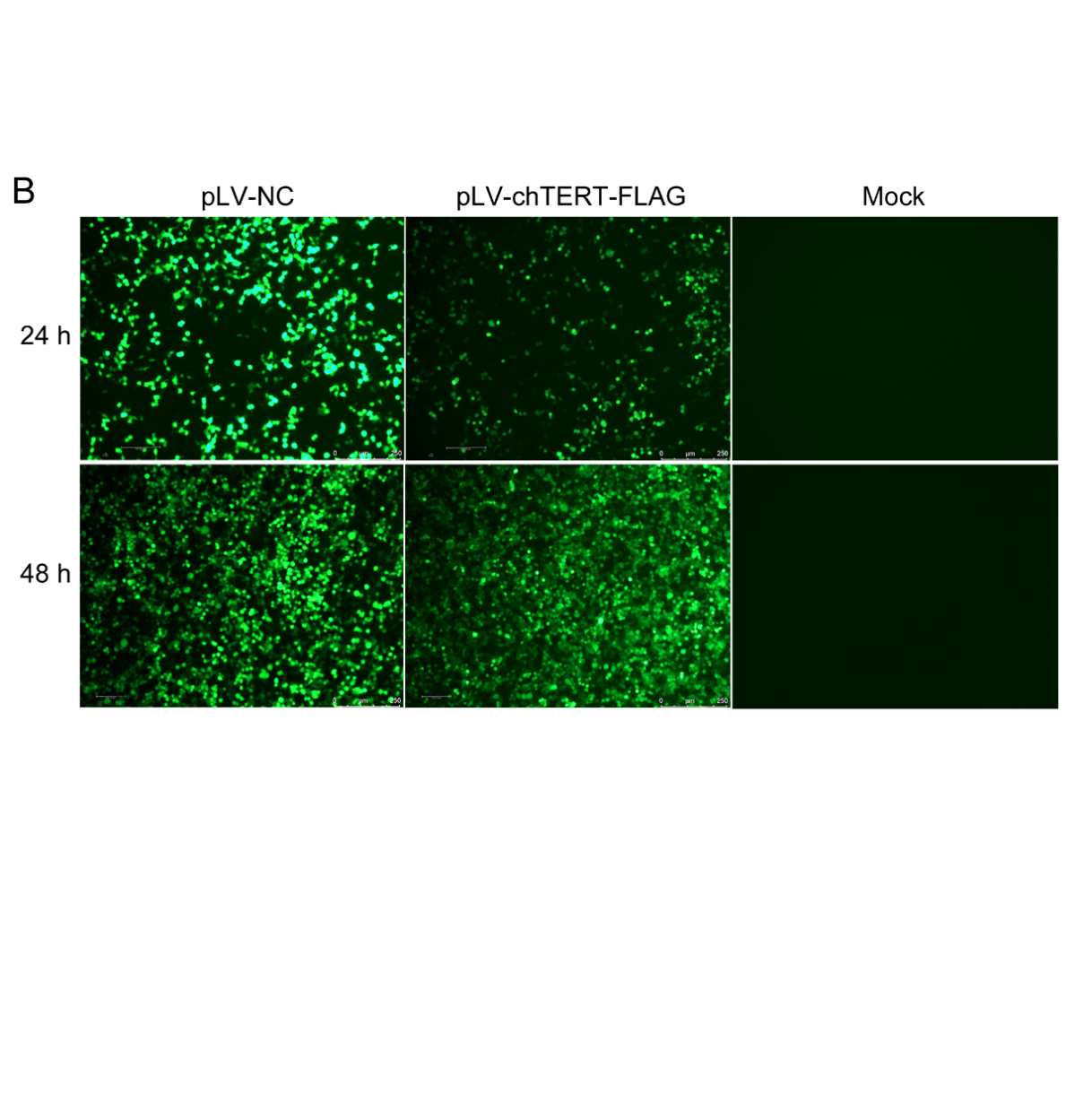

Supplement: Supplementary file 1 — Additional file 1. chTERT protein was successfully expressed in in vitro cells. Observation of LMH cells after transient transfection (A) and 293 T cells after packaging lentivirus, (B) under a fluorescence microscope (×50) [file 13567_2022_1120_MOESM1_ESM.docx]

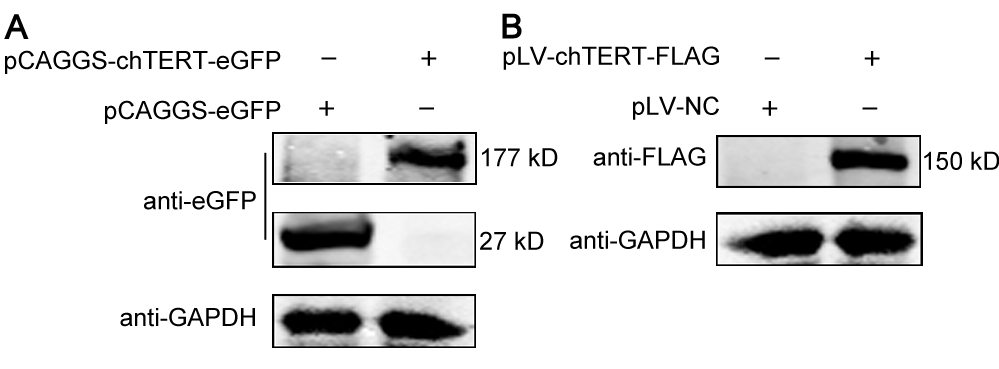

Supplement: Supplementary file 2 — Additional file 2. Western blot analysis of chTERT expression in vitro. (A) chTERT eukaryotic expression plasmid was transfected into LMH cells. (B) Packaging of recombinant lentivirus particles of chTERT in 293 T cells [file 13567_2022_1120_MOESM2_ESM.docx]

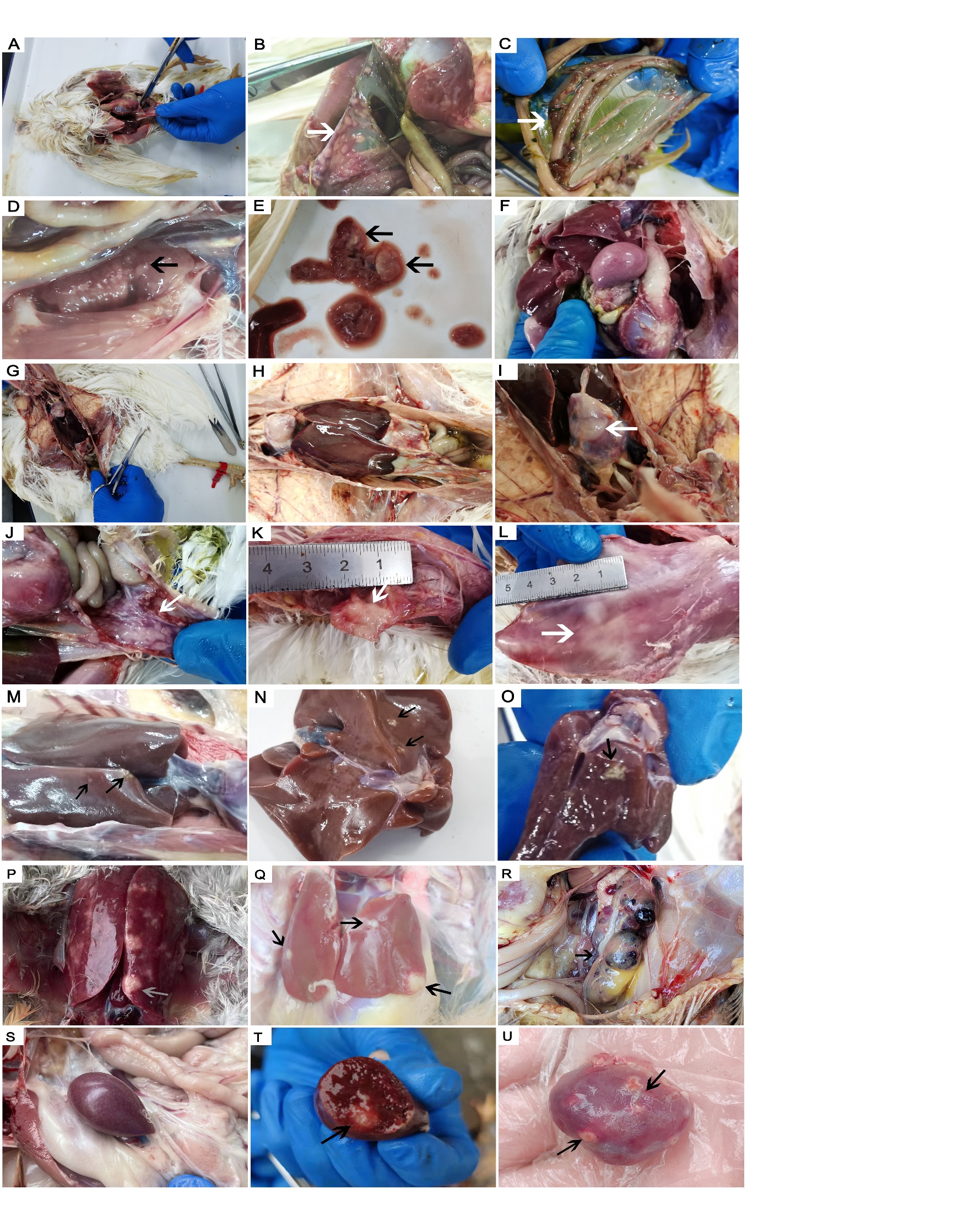

Supplement: Supplementary file 3 — Additional file 3. Pathological autopsy of sick chickens in artificial tumorigenic experiments (A-O) and clinical cases from breeding poultry farms (P-U). During the housing period of the artificial tumorigenic experiment, 3 chickens developed solid tumors (No. 93 (A-F), No. 139 (G-L) and No. 129 (J-O)) at 85 days, 113 days, and 116 days after hatching, respectively. Pathological autopsy revealed typical mesenteric sarcoma (B, C, J), dissection of the kidney revealed grayish white tumor nodules (D, E), thymus enlargement (K), cardiac tumors (I), large grayish white tumor nodules in the pectoral muscle (L) and grayish white tumor nodules in the liver (M, N, O). The livers and spleens of sick chickens I and II were enlarged without tumor nodules (F, H). Moreover, the necropsy of chickens with suspected tumors from clinical farms (P-U) showed prominent grayish white tumor nodules in the liver (P, Q), spleen (S, T, U) and kidney (R). The typical grayish white nodules are indicated by black arrows [file 13567_2022_1120_MOESM3_ESM.docx]

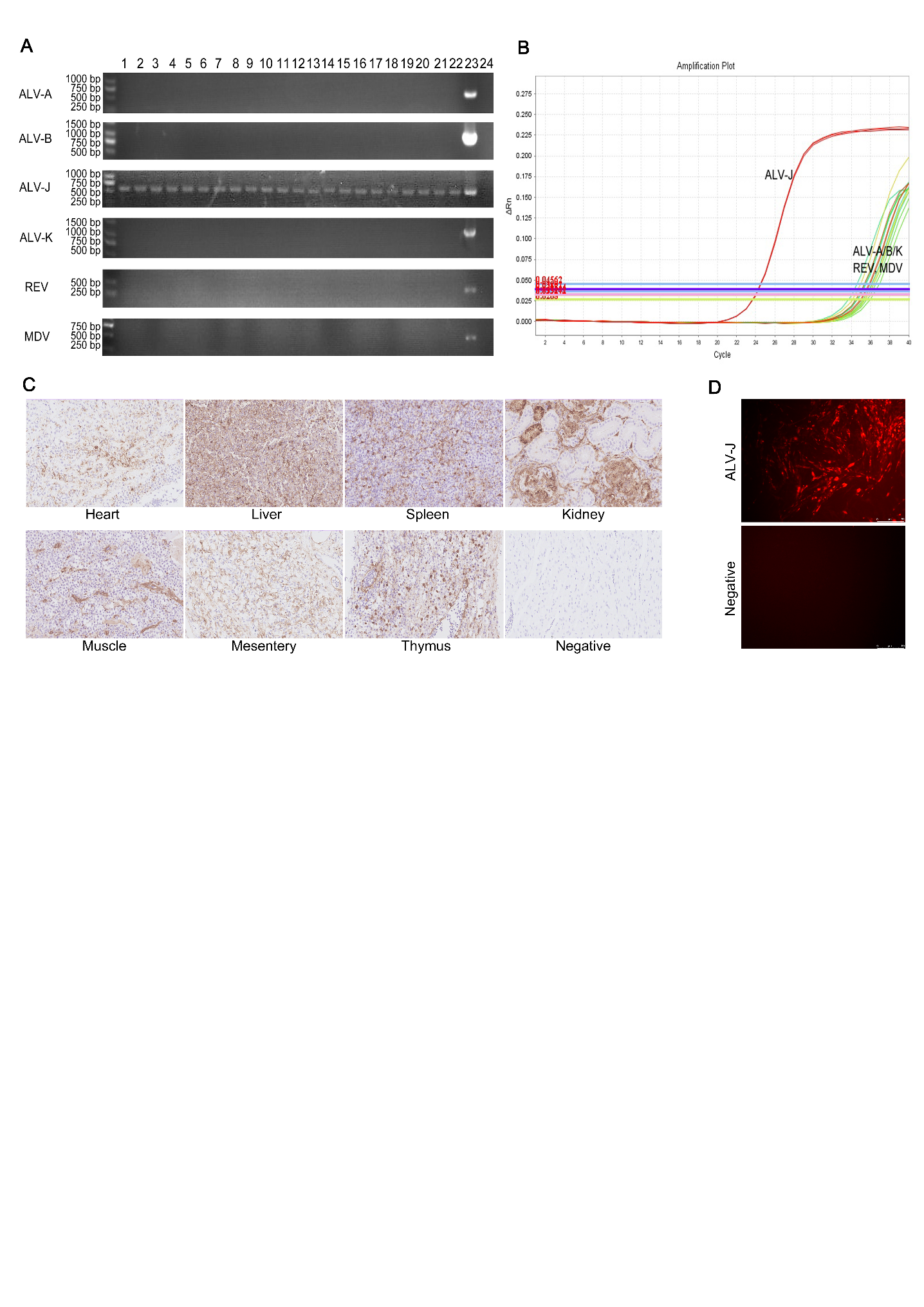

Supplement: Supplementary file 4 — Additional file 4. Isolation and identification of ALV-J. (A) Agarose gel electrophoresis image of PCR products; The results showed that only ALV-J was detected in tumor tissues. lanes 1–22: tumor samples, lane 23: positive control, lane 24: the negative control; (B) The results of RT-qPCR showed that only ALV-J was detected in tumor tissues. (C) Immunohistochemical analysis of ALV-J in tumor tissue (×400); The brown areas are cells that are positive for the target protein (ALV-J gp85). (D) Immunofluorescence staining of ALV-J (×400). ALV-A: avian leukosis virus subgroup A; ALV-B: avian leukosis virus subgroup B; ALV-J: avian leukosis virus subgroup J; ALV-K: avian leukosis virus subgroup K; MDV: Marek’s disease virus; REV: Reticuloendotheliosis virus [file 13567_2022_1120_MOESM4_ESM.docx]

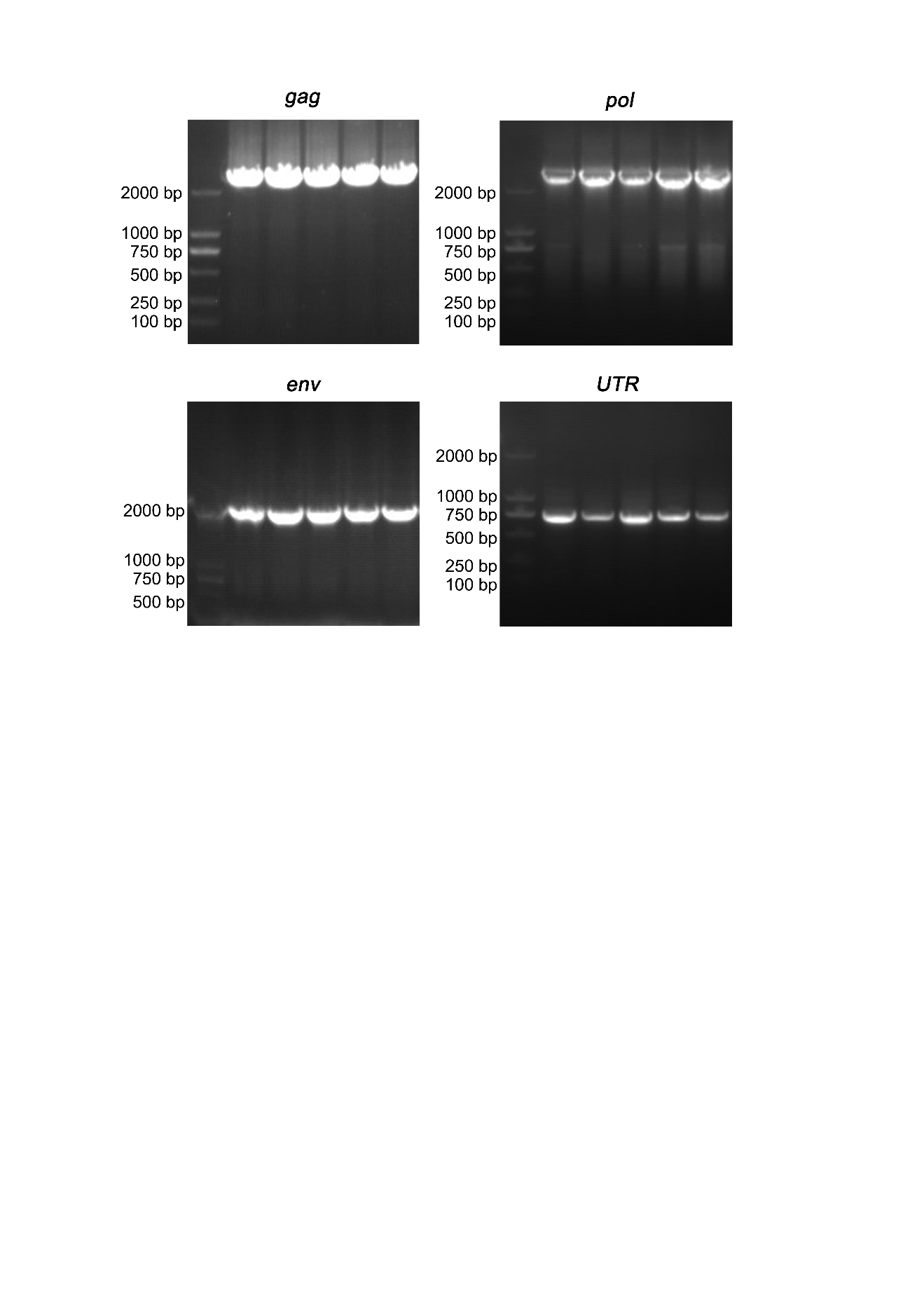

Supplement: Supplementary file 5 — Additional file 5. Agarose gel electrophoresis of the amplification products of the ALV-J genome. The complete genome of chronic transformed ALV-J, including gag, pol, env and UTR, was successfully amplified [file 13567_2022_1120_MOESM5_ESM.docx]

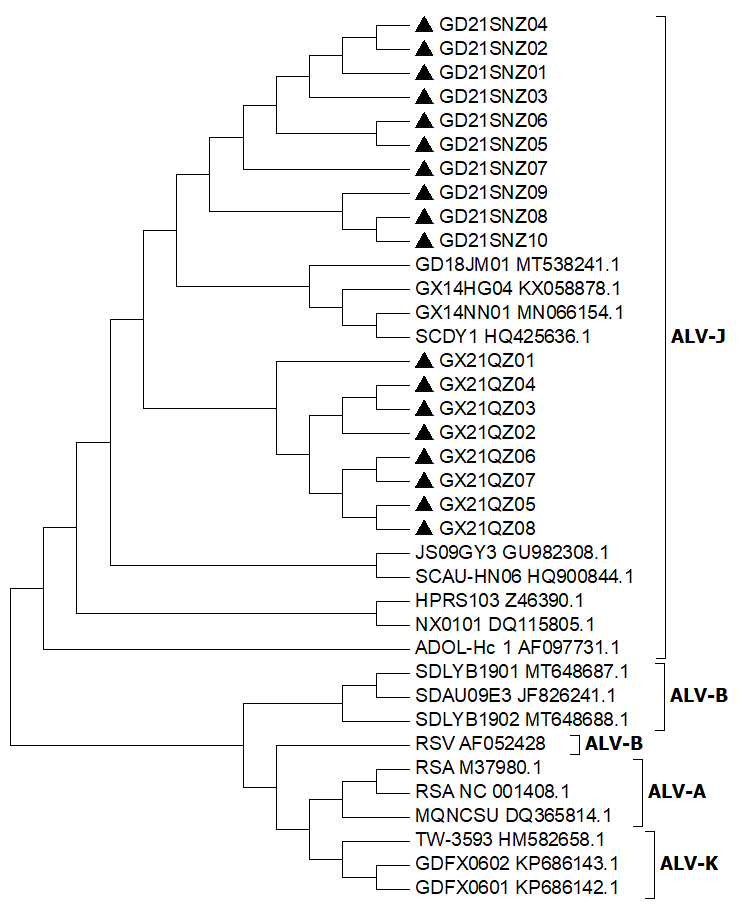

Supplement: Supplementary file 6 — Additional file 6. Genetic evolution analysis of the whole genome (5’- UTR-gag-pol-env-UTR-3’) of ALV-J isolates. “▲” represents the chronically transformed ALV-J strains which were isolated from breeding poultry farms in this study. The results showed that the ALV-J strains isolated from breeding poultry farm were closely related to other classical chronic transformation ALV-J, such as NX0101 and SCAU-HN06. [file 13567_2022_1120_MOESM6_ESM.docx]
