# Supplementary material for: Chicken telomerase reverse transcriptase promotes the tumorigenicity of avian leukosis virus subgroup J by regulating the Wnt/β-catenin signaling pathway
Source: Vet Res. 2022 Dec 2;53:100. doi: 10.1186/s13567-022-01120-2 (PMC9717515; doi:10.1186/s13567-022-01120-2)
Supplement: Supplementary file 7 — Additional file 7. ALV-J tumor tissues and their sources and quantities. [file 13567_2022_1120_MOESM7_ESM.docx]

| Tumor  tissues | Source and quantity of tissue samples ^(1)^ | | Total |
| --- | --- | --- | --- |
|  | Artificial  Tumorigenic | Clinical  Cases |  |
| Heart | 1 | 0 | 1 |
| Liver | 1 | 12 | 13 |
| Spleen | 0 | 4 | 4 |
| Kidney | 1 | 2 | 3 |
| Muscle | 1 | 0 | 1 |
| Mesentery | 2 | 0 | 2 |
| Thymus | 1 | 0 | 1 |
| Total | 7 | 18 | 25 |

^(1)^ tumorigenic means tumors obtained from the ALV-J artificial tumorigenic experiment in this study. Due to the small number of ALV-J tumors obtained from this artificial tumorigenicity experiment, which was not conducive to the development of subsequent experiments. Therefore, another 18 ALV-J tumors were identified and collected from large-scale breeding poultry farms, namely Clinical cases, for further analysis.
